# Cardiorenal Associations in Preclinical Modeling: A Systematic Review and Meta-Analysis

**DOI:** 10.3390/ijms27083477

**Published:** 2026-04-13

**Authors:** Magdalena Jasińska-Stroschein

**Affiliations:** Department of Biopharmacy, Medical University of Lodz, ul. Muszyńskiego 1, 90-151 Lodz, Poland; magdalena.jasinska-stroschein@umed.lodz.pl

**Keywords:** cardiorenal syndrome, heart failure, meta-analysis, renal failure, rodents

## Abstract

Recent years have seen growing interest in the relationship between the heart and kidney disease, resulting in the general term cardiorenal syndrome (CRS) being coined for disorders involving both the heart and kidneys. However, no accurate animal model exists that can replicate the specific cardiorenal associations characteristic of the human CRS subtype. Preclinical studies published between 1990 and 2024 were identified from online electronic databases. These were reviewed and subjected to meta-analysis according to PRISMA, with the quality assessed using the SYRCLE tool. In total, the review and analysis included 251 papers discussing the rodent presentation of cardiorenal associations, expressed by various hemodynamic, echocardiographic and histopathologic parameters, and selected molecular hallmarks. A wide spectrum of invasive and non-invasive animal approaches has been proposed for CRS. Numerous approaches evoked cardiorenal impairments by elevating systemic pressure. Among the “one-hit” models, Dahl/SS and ISO-HF most commonly resulted in cardiac and renal alterations mimicking CRS-2, while DOCA-salt or STZ were the most likely to elicit cardiac injury in progression of renal failure. The clinical relevance of “two-hit” animal models of cardiorenal associations merits another study.

## 1. Introduction

Recent years have seen growing interest in the relationship between the heart and kidney disease. It has been proposed that the complex pathophysiological disorder comprising both the heart and kidneys should be awarded the term cardiorenal syndrome (CRS), in which “acute or chronic dysfunction in one organ may induce acute or chronic dysfunction in the other organ” [1]. Cardiorenal syndrome has been further classified in humans into five clinical phenotypes according to the primary driver of organ dysfunction (type 1 and 2—cardiac; 3 and 4—renal; 5—systemic disease) and chronicity (type 1 and 3—acute; 2 and 4—chronic; 5—acute or chronic) [2]. Clinical and epidemiological observations have confirmed that the coincidence of kidney failure with heart failure (HF) is characterized by poor prognosis, with increased morbidity and mortality [3]. For example, in chronic cardiorenal syndrome type 4 (renocardiac syndrome), chronic renal dysfunction (chronic kidney disease, CKD) results in damage to the cardiac vasculature, and cardiovascular events such as myocardial infarction, sudden death, arrhythmia or cardiomyopathy account for approximately 40% of deaths of patients with end-stage renal disease [4].

To accurately mimic a type of human CRS, an animal model should display the onset of one condition that temporally precedes the occurrence or progression of another (temporal association), and the occurrence of the latter can be mechanistically explained by the former (pathophysiological plausibility) [1]. It is believed that animal models can facilitate the exploration of specific cardiorenal associations at the functional, biochemical and molecular level, with their possible pathophysiological mechanisms. As such, they may be valuable tools for researching disease subtypes and their risk factors, and for identifying candidate drugs for future clinical evaluations.

The present study quantitatively evaluates the extent to which specific rodent models exhibit the onset of one pathological condition followed by the subsequent development of another. It provides a ranked comparison of models according to the breadth of CRS features they display. In addition, it quantitatively assesses the relationships between selected renal and cardiac parameters by meta-regression analyses, and examines the potential influence of experimental covariates on cardiorenal outcomes (primary aim). The study also includes a narrative review of existing rodent models, and provides a brief overview of their mechanisms and their translational relevance to human pathology (secondary aim), thereby aiming to identify models that most comprehensively replicate CRS.

## 2. Methods

This systematic review was conducted according to the Preferred Reporting Items for Systematic Reviews and Meta-Analyses (PRISMA) 2020 statement. The search criteria comprised preclinical experiments on rodents (P = population) that were subjected to a variety of surgical or genetic interventions (I = intervention) to induce renal and cardiac lesions (O = outcome) in comparison to healthy subjects (C = comparator). Only preclinical experiments reporting alterations in at least one “renal” parameter and at least one “cardiac” parameter were included in the analyses. The following were excluded: studies with missing data (e.g., the number of subjects or healthy animal data), in vitro studies, studies performed on isolated organs. Also studies on cardiovascular kidney metabolic (CKM) syndrome [5] were excluded from the analysis (see below).

**Figure 1:**
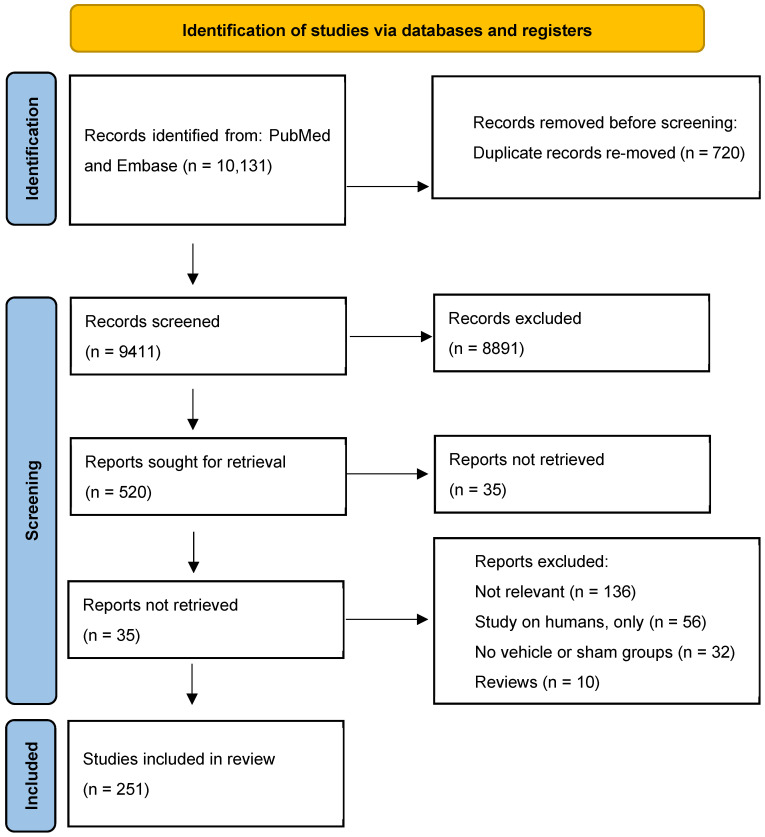
The PRISMA flowchart according to [7].

PubMed, and Embase were searched from January 1992 to December 2024 (Figure 1); the keywords are listed in detail in Supplementary Data. The following data were extracted: animals (species, strain, sex, initial age), model featuring renal and cardiac impairments (surgical interventions; genetic modifications; specific agent—“inducer”; induction period), morphometric, laboratory, hemodynamic, echocardiographic and histopathologic parameters.

The following data were extracted for quantitative analysis: morphometric parameters—body weight (BW), left ventricle (LV) mass, LV weight/tibia length (TL), LV/BW index; heart weight—HW/BW index, kidney mass, kidney mass/BW index; laboratory data—serum creatinine (S-Cre), blood urea nitrogen (BUN), urinary protein excretion ratio (UProtEx), urinary albumin excretion ratio (UAlbEx), urinary albumin to creatinine excretion ratio (ACR), glomerular filtration rate (GFR); hemodynamic data—systolic blood pressure (SBP), left ventricular end-systolic pressure (LVESP), left ventricular end-diastolic pressure (LVEDP), cardiac output (CO), cardiac index (CI), time needed for relaxation of 50% maximal left ventricular pressure to baseline (tau), maximal rate of pressure rise (dP/dtmax), maximal rate of pressure decline (dP/dtmin); echocardiographic data—left ventricular end-systolic diameter (LVESd), left ventricular end-diastolic diameter (LVEDd), left ventricular ejection fraction (EF), fractional shortening (FS), ratio of E-wave to A-wave (E/A); and histopathological data—myocardial fibrosis [interstitial (perivascular) fibrosis, fibrosis area or collagen fraction], and glomerulosclerosis index score (GI), as well as renal fibrosis. Other data concerned inter alia fractional excretion of sodium (FENa%), renal blood flow (RBF), creatine kinase MB (CK-MB), and neutrophil gelatinase-associated lipocalin (NGAL).

The strength of evidence for key outcomes (parameters) was informally graded based on study design, risk of bias, consistency across trials, and precision of effect estimates. Leave-one-out sensitivity analysis was performed to assess the robustness of the results and to identify individual comparisons that may significantly affect the pooled results. Heterogeneity was assessed using Cochran’s Q, with *p* ≥ 0.05 suggesting the absence of heterogeneity. The quality of each individual study was assessed using the SYRCLE risk of bias tool for animal studies [6]. Publication bias across studies was evaluated using Egger’s weighted regression, with *p* > 0.05 suggesting the absence of missing studies, and the Duval and Tweedie ‘trim and fill’ method. Two independent reviewers (MJ-S, KO) searched the literature, extracted data, and assessed the quality of individual studies. Any disagreements were discussed until a consensus was reached. For detailed information, see the Supplementary Data.

The effect size, with a 95% confidence interval (CI), was expressed as the difference in means (D) according to Equation (1),

*D* = *X*(*Model*) − *X*(*Sham*), (1)

or as response ratio (R) according to Equation (2),

*R* = *X*(*Model*)/*X*(*Sham*), (2)

where X is the mean response in the group of animals and Sham is the placebo control group (healthy subjects). As pronounced heterogeneity was expected between studies, a random-effects model was chosen for analysis. At least three independent interventions per parameter and per animal model were required for inclusion in the quantitative analysis. To determine the influence of model-related variables on the final outcome, subgroup analyses (qualitative variables, e.g., animal species, strain) or meta-regression (quantitative variables, e.g., SBP, S-Cre, experimental period, UProtEx, UAlbEx) were performed. A *p*-value below 0.05 was considered statistically significant. The analyses were performed using STATISTICA 13.1 software.

## 3. Results and Discussion

Of the 10,131 titles that were searched, 485 full-text articles were considered to be relevant. Finally, 251 papers were in the systematic review. These comprised 376 interventions, with intervention defined as a separate comparison between vehicle (model) and healthy subjects (placebo control group) according to a particular experimental protocol [8,9,10,11,12,13,14,15,16,17,18,19,20,21,22,23,24,25,26,27,28,29,30,31,32,33,34,35,36,37,38,39,40,41,42,43,44,45,46,47,48,49,50,51,52,53,54,55,56,57,58,59,60,61,62,63,64,65,66,67,68,69,70,71,72,73,74,75,76,77,78,79,80,81,82,83,84,85,86,87,88,89,90,91,92,93,94,95,96,97,98,99,100,101,102,103,104,105,106,107,108,109,110,111,112,113,114,115,116,117,118,119,120,121,122,123,124,125,126,127,128,129,130,131,132,133,134,135,136,137,138,139,140,141,142,143,144,145,146,147,148,149,150,151,152,153,154,155,156,157,158,159,160,161,162,163,164,165,166,167,168,169,170,171,172,173,174,175,176,177,178,179,180,181,182,183,184,185,186,187,188,189,190,191,192,193,194,195,196,197,198,199,200,201,202,203,204,205,206,207,208,209,210,211,212,213,214,215,216,217,218,219,220,221,222,223,224,225,226,227,228,229,230,231,232,233,234,235,236,237,238,239,240,241,242,243,244,245,246,247,248,249,250,251,252,253,254,255,256,257,258].

### 3.1. Quality and Risk of Bias

Figure S1 (Supplementary Material) demonstrates the results of quality assessments for selected studies. They were categorized as having “low risk,” “some concerns,” or “high risk” of bias, with these judgments informing the strength of evidence. Most studies demonstrated a low possibility of missing studies, as confirmed by the trim and fill procedure and the Egger test value (*p* > 0.05) (Table S1). The results of the sensitivity analysis are given in Table S2: mostly, the selected studies did not affect the pooled results for the particular cardiac and renal parameters assessed in the present paper (Supplementary Data).

### 3.2. Characteristics of Protocols Included into Analysis

Of the analyzed experimental protocols (251 papers, 376 interventions with a total number of 5767 animals), the most numerous were surgically subtotal nephrectomized (5/6 SNX) rodents (N = 67/376 interventions; 17.5%), animals subjected to coronary artery ligation (MI [LAD-MI] (N = 64 interventions; 17.0%), unilateral or bilateral renal ischemia–reperfusion injury (N = 27; 7.3%), and Dahl salt-sensitive rats (Dahl/SS) (N = 23; 6.1%). The mean infarct size reported for animals subjected to coronary artery ligation was 41.2% (95%CI 1.53) and 31.3% (95%CI 5.01) in a model combining coronary artery ligation with subtotal nephrectomy (5/6 SNX + MI [LAD-MI]). For more detailed information, see the Supplementary Material (Table S3). The approaches intended to model cardiovascular kidney metabolic (CKM) syndrome, illustrating the condition with a significant contribution of metabolic disorders leading to obesity and diabetes [5]. They can demonstrate partial overlap with CRS (e.g., leptin- and leptin receptor-deficient rodent models). Nevertheless, due to the lack of relevant data—specifically the absence of both “cardiac” and “renal” parameters—studies on CKM syndrome were excluded from the analysis.

For the most common rodent models combining renal and cardiac features, the following are presented (Table 1): changes in systolic function and morphology (dP/dtmax, EF, FS, LVESP, LVESd), diastolic function and morphology (LVEDP, dP/dtmin, LVEDd), myocardial hypertrophy (LV mass, LV/BW ratio, heart weight, HW/BW), myocardial fibrosis and systolic blood pressure (SBP). The table also summarizes the alterations in renal hypertrophy and fibrosis, glomeruloslerosis index, and the laboratory parameters BUN, S-Cre, UProtEx, UAlbEx, ACR, and GFR, as well as selected subcellular hallmarks of cardiac and renal injury or molecular circuits linking cardiac and renal injury, as identified through single-nucleus RNA sequencing (snRNA-seq) and proteomic analyses. A more detailed quantitative analysis according to individual parameters and models is presented in the Supplementary Material (Table S4).

The tree plots reveal pronounced heterogeneity between individual animal approaches according to renal and cardiac impairments. The most significant worsening of systolic function or morphology, expressed as a decrease in left ventricle ejection fraction (EF), fractional shortening (FS), maximal rate of pressure increase (dP/dtmax), left ventricle end-systolic pressure (LVESP) or as an increase in left ventricular end-systolic diameter (LVESd), was noted in animals with isoproterenol-induced HF (ISO-HF), transverse aortic constriction (TAC), aorto-caval fistula (ACF) and coronary artery ligation.

Animals subjected to isoproterenol-induced HF (ISO-HF), transverse aortic constriction, or coronary artery ligation demonstrated the most pronounced worsening in diastolic function and morphology, indicated by an increase in left ventricle end-diastolic pressure (LVEDP) or left ventricle end-diastolic diameter (LVEDd), and a decrease in maximal rate of pressure decline (dP/dtmin).

Renal insufficiency was mainly noted among subtotal nephrectomized subjects (5/6 SNX), DOCA-injected uninephrectomized ones, and Dahl salt-sensitive rats. This insufficiency was expressed as an increase in renal laboratory parameters: serum creatinine, albumin urinary excretion, protein urinary excretion, albumin to creatinine excretion ratio (ACR), or renal fibrosis.

**Figure 2:**
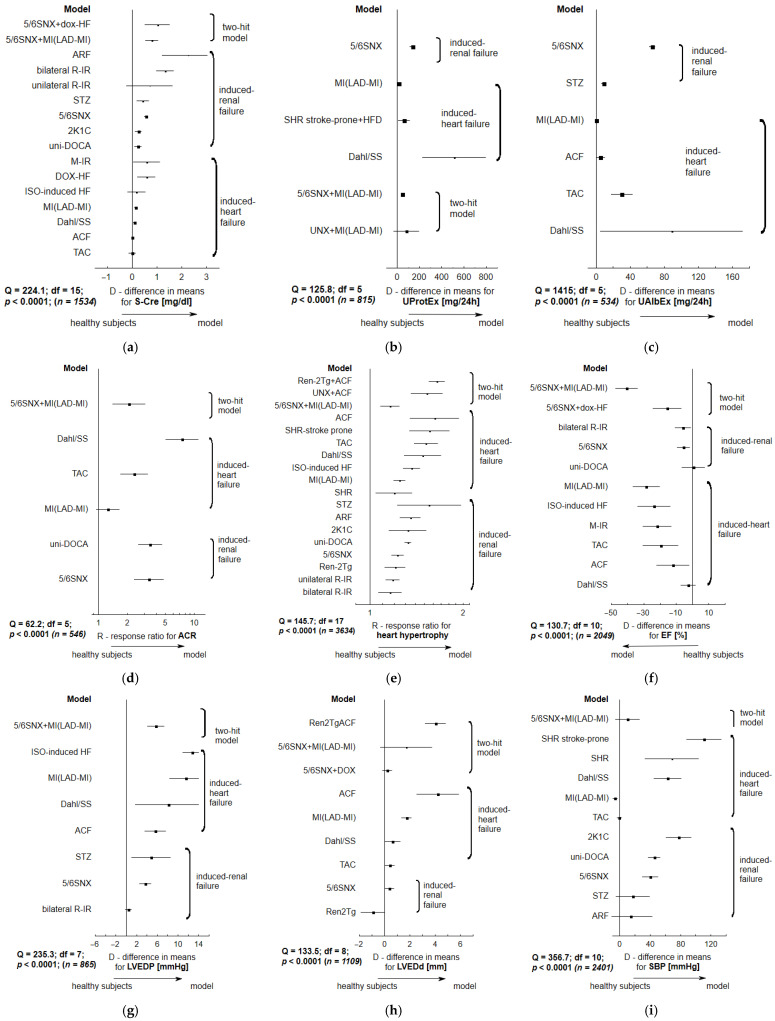
The tree-plots for alterations in renal and cardiac features in selected CRS animal models. (**a**) serum creatinine (S-Cre, mg/dL); (**b**) urinary protein excretion ratio (UProtEx, mg/24 h); (**c**) urinary albumin excretion ratio (UAlbEx, mg/24 h); (**d**) urinary albumin to creatinine excretion ratio (ACR); (**e**) cardiac hypertrophy; (**f**) left ventricular ejection fraction (EF, %) as a primary measure of systolic dysfunction; (**g**) left ventricular end-diastolic ratio (LVEDP, mmHg) as an indicator of left ventricular diastolic dysfunction; (**h**) left ventricular end-diastolic diameter (LVEDd, mm) as an indicator of stiffness (impaired relaxation); (**i**) systolic blood pressure (SBP, mmHg). Effect size was expressed as difference in means (D) (±95% confidence interval—CI) or response ratio (R) (± 95% CI). *p* < 0.05 for Q measure indicates the significant heterogeneity between animal models; *n*—total number of tested animals. The direction of the arrow indicates a trend toward worsening renal (or cardiac) parameters. More detailed results are given in the Supplementary Data (Table S4).

In general, CRS types 3 and 4 displayed more significantly marked renal lesions. CRS types 1 and 2 presented numerous signs of cardiac failure; these signs were more pronounced than those noted in renal injury models alone (CRS-3 and 4). The tree plots indicate that different models of induced-heart failure demonstrated varying potential to develop renal impairments (CRS types 1 and 2), as did models of induced-renal failure to develop cardiovascular consequences (CRS types 3 and 4). In some cases, the provoked alterations were only slightly marked (Figure 2).

**Figure 3:**
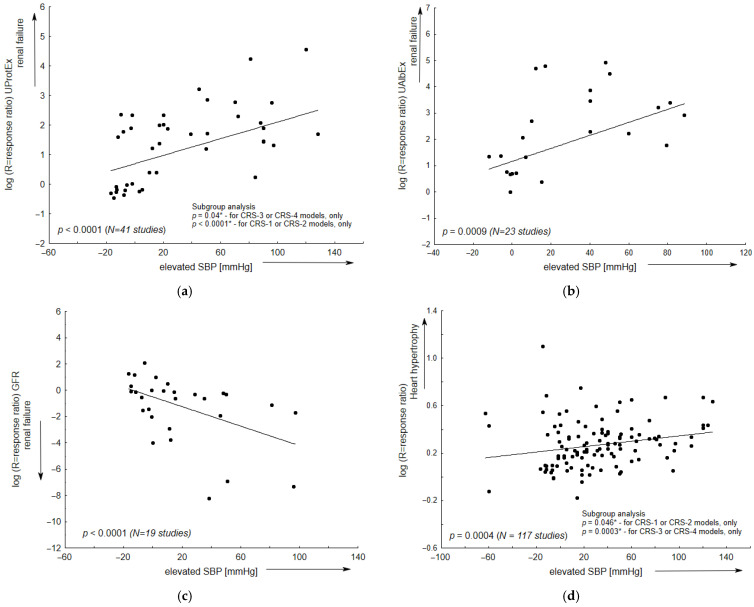

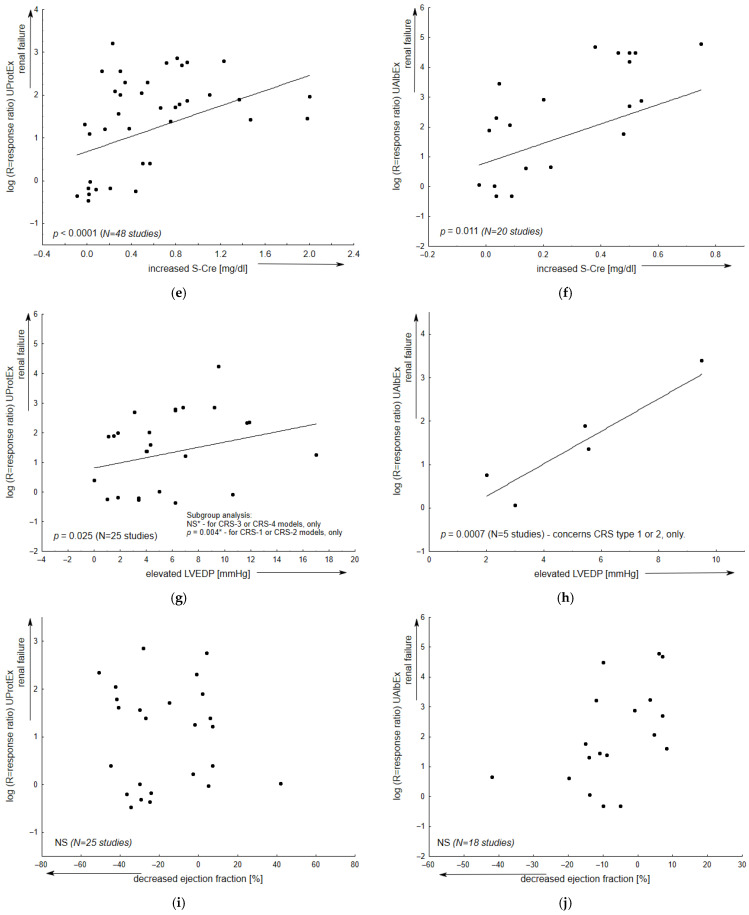
The relationship between renal and cardiac parameters in selected CRS animal models. Meta-regression lines have been fitted to indicate effect size expressed as response ratio (R). (**a**–**d**) Systolic blood pressure (SBP, mmHg); (**e**,**f**) (S-Cre, mg/dL); (**g**,**h**) left ventricular end-diastolic pressure (LVEDP, mmHg) as an indicator of left ventricular diastolic dysfunction; (**i**,**j**) left ventricle ejection fraction (EF,%) as a primary measure of systolic dysfunction; N—total number of comparisons. The arrow direction indicates the trend toward worsening renal (or cardiac) parameters. The meta-regression results for all models are given; in Panels (**a**,**d**,**g**), the data includes the outcomes of the subgroup analyses (*) based on data from cardiorenal (CRS types 1 and 2) or renocardiac (CRS types 3 and 4) models. For example, a significant correlation (*p* < 0.001) between diastolic dysfunction—reflected by elevated LVEDP—and proteinuria (UProtEx) was observed in CRS types 1 and 2, but not in CRS types 3 and 4.

The most pronounced correlations between individual cardiac and renal parameters in the selected animal models are presented in the meta-regression plots. These correlations included elevated systolic blood pressure, which was related to decreased glomerular filtration rate (GFR), and increased albuminuria, proteinuria, or myocardial hypertrophy; they were also characterized by increased serum creatinine, which was associated with decreased GFR, or increased UProtEx and UAlbEx. In addition, elevated left ventricle end-diastolic pressure was associated with increased UProtEx, UAlbEx, as well as with myocardial hypertrophy (Figure 3).

In addition, animal age, species, strain and duration of experimental period were found to have a pronounced influence on cardiac outcomes. In addition, in models mimicking CRS types 3 or 4, most of the studied renal parameters (ACR, UProtEx, UAlbEx, GFR, renal fibrosis) worsened as the length of the investigation period increased (Supplementary Figure S2).

The present findings demonstrate considerable variation between the reviewed surgically driven, genetic and drug-induced animal models aimed at mimicking human cardiorenal associations. The approaches exhibited varying potential to provoke renal and cardiac disturbances; they also influenced a wide spectrum of hemodynamic, echocardiographic, and histopathologic parameters to varying degrees, as well as a number of varied laboratory markers. They hence demonstrate potential for further preclinical and clinical experiments.

### 3.3. Animal Models for Cardiorenal Syndrome Type I and II

Hence, the prognosis of heart failure remains poor, particularly when it is accompanied by kidney dysfunction. For many years, research into cardiorenal syndrome has employed small animal models in which both ischemic injury-induced HF (due to acute myocardial infarction) and non-ischemic injury-induced HF have been provoked: the latter by chronic pressure overload. These models are then exacerbated by subsequent acute renal impairments to replicate cardiorenal syndrome (CRS) type 1 [259].

In contrast, in CRS type 2, the occurrence or progression of chronic kidney disease is preceded by chronic HF. Such cases are characterized by abnormalities in kidney structure or function, expressed by albuminuria (ACR ≥ 30 mg/g), urine sediment abnormalities, persistent hematuria, electrolytes and other abnormalities due to tubular disorders, abnormalities detected by histology, structural abnormalities detected by imaging, as well as decreased GFR (GFR < 60 mL/min/1.73 m^2^) present for at least three months [260]. Renal fibrosis can be strongly correlated with the development of chronic kidney disease, leading to hypertension, anemia and electrolyte disturbance [1].

When choosing an animal model of acute cardiac event (e.g., acute myocardial infarction) for modeling CRS type 2, it is important that the selected model facilitates the development of chronic left ventricular (LV) dysfunction, followed by the onset, or progression, of chronic kidney disease [1]. For both CRS types 1 and 2, it is crucial to assess the effects of cardiac injury models on kidney function. For example, in the case of CRS-1, acute heart injury can result in reduced cardiac output, leading to reduced renal filtration and renal blood flow; this pathway is believed to serve as the “cardiorenal connector”. Inflammatory signaling, venous congestion, endocrine signals and sympathetic activation have been proposed as other regulators of the interaction between heart and kidney function [259].

Ischemia-induced heart injury. The most common model used for exploring CRS type 1 and type 2 was coronary artery ligation [1,259]. For type 2, the surgical procedure did cause some permanent heart failure. The papers (N = 28 studies) discussed the cardiovascular effects of treatment; these included myocardial hypertrophy (*p* < 0.0001) and fibrosis, followed by the onset of heart failure with reduced ejection fraction (EF: 39.2, 95%CI 31.2–47.2%, *p* < 0.0001) accompanied with reduced LVESP and pronounced diastolic dysfunction (↑LVEDP and LVEDd, *p* < 0.0001). The renal failure manifested with microalbuminuria and proteinuria (mean increase in UProtEx by 12.5 mg/24 h, *p* = 0.005), as well as a mild increase in serum creatinine and renal fibrosis. These alterations were accompanied by an increase in markers of kidney disease progression (NGAL, cystatin C) and inflammation (Il-6 and Il-beta1). However, the model can induce extensive infarction and rapid structural remodeling, resulting in high heart failure-related mortality. It can also be a source of substantial variability in experimental outcomes.

Pressure overload-induced non-ischemic heart injury. The rodent transverse aortic constriction (TAC) model is characterized by non-ischemic injury-mediated heart failure caused by pressure overload; it is mainly recognized as a chronic pressure-overload model (CRS type 2) [250]. This procedure has been found to not affect systemic hypertension; therefore, it is unclear how it may alter renal function. The signs of cardiac injury can include systolic dysfunction (↓EF and FS, *p* < 0.0005), impaired systolic morphology (↑LVESd, *p* = 0.04) and myocardial hypertrophy (*p* < 0.0001), as confirmed in the current analysis. In individual studies, plasma urea concentration, a surrogate marker for renal dysfunction, was found to be elevated post-TAC. Increased plasma renin concentration was also reported, which could reflect hypoperfusion of the kidney. Present synthesis of previous studies indicates that TAC can result in a roughly two-fold increase in albumin-to creatinine ratio (*p* < 0.0001). However, no structural abnormalities or kidney hypertrophy were present. Therefore, to overcome these limitations, further studies are needed to explore the bidirectional interactions between the heart and kidney in the TAC model.

Aorto-caval fistula (ACF) presents a well-established model of HF caused by volume overload; it results in activation of vasoconstrictor/antinatriuretic neurohormonal systems (e.g., renin–angiotensin–aldosterone system (RAAS)), sodium retention and impairment of renal function—fundamental drivers of CRS-2 pathophysiology congestion. ACF has been recommended by the American Heart Association and the European Society of Cardiology for preclinical testing to identify new targets for the treatment of HF patients [261]. ACF can be used as part of a “two-hit” approach, combined with Ren-2 transgenic rats (TGR): in this case, the transgenic rat model incorporates the mouse Ren-2 renin gene [TGR; strain name TGR(mRen2)27], which presents angiotensin II (Ang II)-dependent hypertension. The model has been found to be particularly effective at exploring cardiorenal interaction in the pathophysiology of heart failure [262]. Current analysis confirms that while this “two-hit” model promotes cardiac lesions (cardiac hypertrophy or systolic dysfunction), the renal consequences were poorly expressed. Further studies on his model are required.

Systemic hypertension-mediated heart injury. The salt-sensitive Dahl rat HF model (Dahl/SS) promotes an increased aldosterone level that contributes to poorer kidney function through increased oxidant stress and TGF-β production. Dahl salt-sensitive rats are considered a non-surgical CRS type 2 model, in which chronic HF is followed by renal dysfunction in a predictable, progressive manner. The latter effects are believed to develop via salt-sensitive hypertension and remodeling of the left ventricle. The model also captures the key mechanisms of human CRS-2 (RAAS activation, oxidative stress, fibrosis). It was found to demonstrate an excessive rise in systemic pressure with a mean elevation of 62.7 mmHg (±9.25) compared to healthy subjects (*p* < 0.0001) in the present analysis; this determines the progression of systolic dysfunction and LV hypertrophy [263]. Present data indicate that renal injury manifests with massive albuminuria and proteinuria (≈500 mg/24 h) (*p* < 0.05), ≈ 7-fold increase in ACR (*p* < 0.0001), glomerulosclerosis or renal fibrosis (*p* < 0.0001).

The spontaneous hypertensive rat (SHR) model is a genetic and systemic hypertensive model with a pronounced increase in systolic blood pressure. The kidney injury in this model was suggested to indirectly result from heart failure; as such, SHRs have been proposed as candidates for both CRS-2 and CRS-5 [259]. In the latter approach, concomitant HF and kidney failure can occur together in response to systemic hypertension. Present data confirm that the SHR model can develop significant hypertension (average elevation of SBP by 70 mmHg, *p* = 0.0002), cardiac fibrosis (*p* < 0.0001) and hypertrophy (*p* = 0.009), as well as kidney hypertrophy (*p* < 0.0001). Recent works also emphasize the need to evaluate the cardiorenal associations of stroke-prone spontaneously hypertensive rat (SHR-SP) models. SHR-SP is a unique genetic model of severe hypertension and cerebral stroke. Churchill et al. (2022) report that SHR-SP kidneys are more susceptible than SHRs to renal damage when exposed to the same blood pressure and metabolic environment [264]. Studies have found SHR-SP to be a validated model of malignant, systemic hypertension; it is characterized by severe, sustained high blood pressure affecting multiple organ systems, intrinsic susceptibility to renal injury, and systemic RAAS dysregulation, leading to simultaneous cardiac and renal damage [15,90]. Hence, there is a strong mechanistic basis for using SHR-SP rats to study cardiorenal syndrome type 5, in which a systemic pathological driver, such as malignant hypertension, simultaneously injures both the heart and kidneys [259]. Present findings initially confirm the potential of SHR-SP animals to develop pronounced hypertension (≈110 mmHg, *p* < 0.0001) accompanied by cardiac hypertrophy (*p* < 0.0001) and fibrosis (*p* = 0.002), while subsequent renal impairment was manifested mainly by proteinuria (*p* = 0.02).

Cardiomyopathy-induced model. Cardiomyopathy can be induced by acute models, which mimic stress-induced cardiomyopathy. Chronic models can be used to provoke transition from cardiac compensatory hypertrophy to decompensate heart failure, and subsequent renal failure mediated with increased RAAS activity [16]. In the ISO-induced chronic model, an implanted minipump continuously delivers isoproterenol (ISO), a nonselective β-adrenergic receptor agonist. The method provides sustained β-adrenergic stimulation, which is believed to effectively provoke the development of cardiorenal syndrome type 2 [251]. Present data indicates that the models entail a wide variety of cardiac consequences including myocardial hypertrophy, systolic and diastolic dysfunction. Systolic dysfunction was expressed by a significant decrease in EF (*p* < 0.0001), but not below a threshold of 50%, and in decrease in LVESP (*p* < 0.0001). Diastolic dysfunction was expressed by increase in LVEDP (*p* < 0.0001). The alterations in LVESP and LVEDP were comparable with those produced by coronary artery ligation; however, ISO yielded more pronounced renal fibrosis than the myocardial infarction model. The ISO model can exhibit a range of renal alterations including kidney hypertrophy and kidney fibrosis, which were found to be approximately six-fold higher compared to healthy subjects (*p* < 0.0001). Substantial variability in ISO dosing has been reported, with regimens ranging from 5 to 250 mg/kg. Among the reviewed protocols, the most commonly employed dose was 5 mg/kg per day, administered subcutaneously or intraperitoneally. This is in line with recent findings demonstrating that low-dose subcutaneous ISO administration (5 mg/kg/day) provides a robust and reproducible model of stable heart failure with 100% survival, whereas higher doses (e.g., 60 mg/kg) are associated with increased mortality and marked inter-individual variability [265].

### 3.4. Animal Models for Cardiorenal Syndrome (CRS Type 3 and 4, Renocardiac Syndrome)

Most experimental studies have explored the potential of animal models to reflect chronic kidney disease resulting in subsequent chronic heart failure (CRS, type 4). Chronic kidney disease increases sympathetic activity and activates the RAAS, with subsequent chronic inflammation and oxidative stress, sodium and water retention, reduced availability of nitric oxide, systemic vasoconstriction and glomerular filtration reduction. These pathophysiological mediators have been found to promote cardiac disease in response to kidney failure, e.g., Ang II and reactive oxygen species can induce particularly left ventricular remodeling and congestive heart failure [266]. As a consequence, in animal models intended to mimic CRS type 3 or 4, the documented renal impairment should precede the occurrence or progression of heart failure, with preserved or reduced EF, as well as ventricular hypertrophy, diastolic dysfunction, and increased risk of adverse cardiovascular events [266].

Subtotal nephrectomy (5/6 SNX) model, a gold standard for CKD, combines left partial nephrectomy (2/3) with subsequent total right nephrectomy [142]. It has been the most popular approach for replicating chronic kidney disease with subsequent cardiovascular consequences, with 67 studies recorded in the present analysis. A wide spectrum of renal abnormalities has been reported for this model with the most pronounced being a ≈ 3-fold increase in ACR (*p* < 0.0001), ≈ 2.5-fold decrease in GFR (*p* = 0.003), or a daily mean increase of 65.7 ± 1.8 mg albuminuria (*p* < 0.0001), as compared to healthy subjects. Importantly, animals could develop cardiac disturbances with myocardial fibrosis (≈2.4-fold increase, *p* < 0.0001) and heart hypertrophy (*p* < 0.0001). Subtotal nephrectomy in this model could also lead to heart failure with preserved ejection fraction; the present analysis indicates a slight decrease in EF, but it remains above a threshold of 40% (68.4, 95%CI 64.2–72.6; *p* < 0.0001). Diastolic dysfunction was manifested by a moderate but significant elevation in left ventricle end-diastolic pressure (*p* < 0.0001). These changes were accompanied by increased levels of angiotensin II, natriuretic peptides, and a wide spectrum of molecular hallmarks of inflammation (TNF-alpha, Il-6, Il-beta1, MMPs), and proliferation (TGF-beta). Interestingly, subtotal nephrectomy models demonstrate notable inter-strain variability, with kidney and cardiovascular consequences being more pronounced in 129/Sv than in C57BL/6JRj mice. In addition, the 129/Sv mice appear to be more susceptible to the cardiovascular consequences of CKD than the C57BL/6JRj mice, possibly due to impaired acetylcholine-induced vasorelaxation in the mesenteric artery, caused by reduced acetylcholine receptor expression in abdominal vessels, compared with C57BL/6JRj mice [267]. This phenotypic difference is an important consideration when selecting an appropriate model for studying cardiovascular and renal outcomes in CRS research. Unfortunately, as most of the reviewed experiments were conducted using C57BL/6J mice, no further inter-strain comparison was possible due to insufficient sample sizes.

The combination of subtotal nephrectomy with coronary artery ligation, as proposed in some recent studies, was found to successfully trigger cardiac disturbances toward more pronounced systolic dysfunction (mean EF%: 34.0, 95%CI 28.4–39.5, *p* < 0.0001), and impaired systolic morphology (↑LVESd, *p* = 0.03) as well as diastolic dysfunction (↑LVEDP, *p* < 0.0001). Renal dysfunction was manifested by a significant increase in serum creatinine (*p* < 0.0001), proteinuria (*p* < 0.0001) and renal fibrosis (*p* = 0.0003). Models incorporating coronary artery ligation, either before or after nephrectomy, combine CKD with induction of acute myocardial infarction; such “two-hit” approaches have been proposed to correspond with human CRS type 2 [20] or CRS type 4 [21]. However, the value of the model needs to be confirmed by further research.

Similarly, some studies suggest that the combination of 5/6 subtotal nephrectomy and dilated cardiomyopathy induced by doxorubicin may facilitate the onset of cardiac failure with systolic dysfunction and renal impairment. A well-established “one-hit” animal model is doxorubicin-induced cardiomyopathy, promoting congestive heart failure with diastolic dysfunction. It can be mediated by many factors and pathways such as inflammatory cytokines, oxidative stress, mitochondrial damage, intracellular Ca2+ overload, iron-free radical production, DNA-damage pathways, and myocyte membrane injuries [268]. However, further studies are needed to confirm the utility of a doxorubicin-based “two-hit” approach in modeling cardiorenal associations.

Hypertension-mediated renal injury. The combined administration of deoxycorticosterone acetate (DOCA) with a high-salt diet and unilateral nephrectomy is a well-established model for induction of hypertension-mediated renal injury. The model recreates the hemodynamic, structural, molecular, and inflammatory pathways through which CKD drives chronic cardiac dysfunction. As such, it is regarded as an excellent experimental platform to study CRS-4 [218]. Present findings indicate that the systolic blood pressure was elevated by a mean value of 45.4 ± 4.1 mmHg (*p* < 0.0001) compared to healthy subjects. The procedure was found to achieve glomerulosclerosis and kidney hypertrophy (*p* < 0.0001) or renal fibrosis (*p* = 0.002), accompanied by abnormal laboratory results (↑S-Cre and ACR, *p* < 0.005) in uninephrectomized DOCA subjects. This model mimics aldosterone overload and volume-dependent hypertension, and thus it is also considered a model of human primary aldosteronism [269]. Present data confirm the development of cardiovascular consequences, such as cardiac fibrosis (≈4-fold increase, *p* < 0.0001) and slight myocardial hypertrophy in this model. These alterations were accompanied by elevated natriuretic hormone and increases in markers of kidney disease progression and inflammation (MCP-1, Il-6, KIM-1). Other quantitative analyses found that DOCA-salt can provide an opportunity to mimic human heart failure with preserved ejection fraction (HFpEF) by inducing diastolic dysfunction with pulmonary congestion while preserving systolic function [263]; however, these works did not examine the possible coexistence of renal failure with cardiovascular complications.

The 2K1C model is a rat model of renovascular hypertension, in which one renal artery is constricted to chronically reduce renal perfusion, while the other kidney remains untouched [270]. 2K1C subjects are characterized by an imbalance in the RAAS, characterized by increasing angiotensin II and aldosterone concentration, which can provoke progressive kidney injury and secondary cardiac effects. These mechanisms overlap strongly with the pathophysiological features of CRS type 4, i.e., cardiovascular dysfunction driven by chronic kidney disease. The present synthesis of results from seven studies confirms the potency of this approach to develop pronounced hypertension (SBP elevated by 77.5 ± 8.5 mmHg, *p* < 0.0001) with consequent myocardial hypertrophy and increased natriuretic hormones. Some papers also reported diastolic dysfunction, expressed as an increase in ventricular diastolic eccentricity index (LVEId): a measure of the amount of distortion of ventricular septal geometry due to elevated diastolic pressures or volumes [26]; however, further studies are needed to confirm the extent of cardiovascular consequences in this model.

Similarly, more research is needed to evaluate the potential of adenine-induced chronic renal failure (ARF) in the modeling of cardiorenal associations. In contrast to modalities such as 5/6 nephrectomy, the adenine-based approach is non-surgical and non-invasive. However, its capacity to induce moderate or advanced CKD within a CRS type 4 framework likely depends on both the administered adenine dose and the duration of exposure [271]. Rodents exposed to diets containing 0.075–0.75% adenine or intraperitoneal injections of adenine solution for several weeks can develop chronic kidney disease with renal crystallization and tubulointerstitial fibrosis due to deposition of 2,8-dihydroxyadenine crystals in the kidney. At the molecular level, progression of kidney injury was expressed by increased KIM-1, MCP-1 and plasma urea. In most experiments, such renal impairments have led to cardiovascular lesions manifested by myocardial hypertrophy (*p* < 0.0001). Only a couple of studies have reported failure of systolic or diastolic function or morphology [28]; as such this data cannot provide a conclusive answer when discussing the utility of the model to reflect human renocardiac syndrome.

Ischemia-induced renal injury. Unilateral or bilateral renal ischemia–reperfusion models (R-IR) can clinically reflect ischemic injury in acute kidney disease or early chronic kidney disease, with subsequent cardiovascular consequences. Therefore, R-IR models have been proposed mainly for experiments concerning CRS type 3 (CRS-3), where sudden worsening of cardiac function appears secondary to acute kidney injury (AKI) [29]. Other authors have used bilateral renal ischemia–reperfusion models to provoke transition from AKI to chronic kidney disease, and to investigate heart alterations during progression of chronic kidney disease (CRS type 4) [30]. In both scenarios, i.e., CRS type 3 or 4, the cardiac manifestations included slight myocardial hypertrophy and systolic dysfunction related to a decrease in EF% (average by 5.9 ± 2.4%, *p* = 0.02, as compared to healthy subjects). The model is characterized by a decrease in antioxidant capacity (↓SOD), inflammation (↑TNF-alpha, Il-6) and vasoconstriction (↓eNOS); however, it also demonstrates high variability, risk of acute animal death, and requires careful surgical technique.

Streptozotocin-induced diabetic nephropathy. Another non-invasive model that can result in renal failure, through a different mechanism than systemic hypertension-mediated approaches (e.g., DOCA-salt, 2K1C), is the streptozotocin-based model. Streptozotocin is an irreversible cytotoxic agent that primarily induces type 1 diabetes mellitus (DM) by targeting pancreatic β-cells, followed with subsequent nephropathy [272]. As diabetic nephropathy is one of the most common causes of CKD, it is also one of the most frequent underlying conditions producing CRS type 4. A wide range of doses and dosage regimens are used, which can result in heterogeneous glycemic responses to STZ, i.e., mild, severe or fatal hyperglycemia. Present findings indicate that renal injury manifested with pronounced kidney hypertrophy (≈1.7-fold, as compared to healthy subjects, *p* < 0.0001), renal fibrosis (≈2.4-fold, *p* = 0.0001) or increased serum creatinine level (mean increase in 0.43 ± 0.12 mg/dL, *p* = 0.005). Heart failure can be manifested with cardiac hypertrophy and fibrosis (*p* < 0.005), systolic dysfunction (↓FS%, ↓dp/dtmax, *p* < 0.001), diastolic dysfunction (↑LVEDP: mean increase in 4.8 ± 1.9 mmHg, *p* < 0.05), and increased cardiac troponins, natriuretic hormones and CK-MB. These cardiovascular consequences can develop secondary to renal impairment as well as to diabetes mellitus; the latter is a systemic condition leading to abnormalities of both the cardiovascular and renal systems. Therefore STZ-based model might be suitable for studies exploring CRS type 5, or cardiorenal syndrome associated with diabetes, as proposed by Karnib et al. (2010) [273].

A non-obese, non-hypertensive, spontaneously polygenic model of type 2 diabetes (T2DM) is based on Goto-Kakizaki (GK) rats. The GK rat replicates the characteristic progression of CRS type 4, demonstrating diabetes-induced chronic renal impairment followed by secondary cardiac structural and functional remodeling [274]. This model was generated through repeated inbreeding of glucose-intolerant Wistar rats over multiple generations, resulting in moderate hyperglycemia, peripheral insulin resistance, and a non-hyperlipidemic phenotype. The development of T2DM in GK rats is primarily attributed to a deficit in pancreatic β-cell mass, driven by reduced β-cell proliferation and increased apoptosis. By 24 months of age, i.e., in the later stage of the model, GK rats exhibit albuminuria and glomerulosclerosis, reflecting progressive human diabetic nephropathy [275]. However, as only a limited number of GK experimental protocols were included in the present analysis, the evidence was restricted to single observations without meta-analytic evaluation. Nevertheless, the data indicated a reduction in GFR and increases in ACR, BUN, serum creatinine, and glomerulosclerosis scores, with a median study duration of 11 weeks. The cardiac phenotype reported in these studies consisted primarily of myocardial hypertrophy.

Several studies have proposed that GK rats, or rodents exposed to streptozotocin, might be used to replicate cardiorenal syndrome associated with diabetes [273]. In 2023, the American Heart Association introduced the concept of cardiovascular kidney metabolic (CKM) syndrome; this classification formally defined it as an integrated disease construct in which diabetes contributes to cardiovascular and renal dysfunction through multiple, intersecting biological pathways [5]. The earliest stage is characterized by excess adiposity, possibly with adverse distribution (e.g., abdominal obesity). These abnormalities typically precede the development of type 2 diabetes, hypertension, hypertriglyceridemia (metabolic syndrome), kidney disease and subsequent cardiovascular complications, reflecting the progression of CKM syndrome [276,277]. Taking into account the nature of CKM, where the majority of CKM syndrome factors stem from an excess and dysfunction of adipose tissue, particularly visceral and ectopic body fat, STZ-based models and GK rats are generally not suitable for modeling the integrated, multisystem pathology characteristic of CKM syndrome. No excessive weight gain was observed in these animals. Some rodent approaches (e.g., leptin- and leptin receptor-deficient rodent models) can demonstrate partial overlap with CRS. Nevertheless, due to the lack of relevant data—specifically the absence of both “cardiac” and “renal” parameters—studies on CKM syndrome were excluded from the analysis. To date, no single rodent model accurately replicates the full spectrum of human CKM. Despite ongoing challenges in developing appropriate experimental systems, the problems associated with CKM, like CRS, highlight the growing need for multidisease animal models capable of capturing the complex, interconnected pathophysiology observed in humans [278].

### 3.5. The Relationship Between Cardiac and Renal Impairment

According to the definition of cardiorenal syndrome, an animal model mimicking human CRS should display the onset of one condition that temporally precedes the occurrence or progression of another [1]. In almost half of the models evaluated in the current paper (Dahl/SS, SHR, SHR-SP, DOCA or 2K1C), cardiorenal impairments were intended to be accompanied by systemic hypertension or renovascular hypertension. The present meta-regression analysis demonstrates that elevated systolic pressure was significantly correlated with renal insufficiency, expressed as decreased GFR, and increased albuminuria or proteinuria. This phenomenon applies to separate models of renal failure (CRS type 3, 4) and of heart failure (CRS type 1, 2), as demonstrated in the subgroup analysis. In the latter subgroup (CRS type 1, 2), renal failure could result directly from elevated systolic pressure, as well as from hypertension-mediated myocardial hypertrophy and heart failure. Current analysis also identified a significant relationship between elevated systolic pressure and myocardial hypertrophy (*p* = 0.0004) in the evaluated models. Such hypertrophic lesions were reported for most animal approaches using renal injury as a primary driver, and in various animal models of ischemic or non-ischemic heart injury or isoproterenol-induced heart failure. In addition, diastolic dysfunction was noted in animal models with heart or renal failure as the primary driver. For CRS type 1 or 2, a slight, but statistically significant, relationship (*p* < 0.05) was also found between diastolic dysfunction, expressed by elevated LVEDP, and an increased renal insufficiency, indicated by proteinuria.

## 4. Future Perspectives and Conclusions

Further investigation is needed to assess the utility of specific models and pathogenic pathways such as NG-nitro-L-arginine methyl ester (L-NAME)-induced renal failure and nitric oxide-dependent signaling, and vascular calcification models such as adenine-induced renal injury. The same limitation pertains to “two-hit” approaches, such as the combination of 5/6 nephrectomy (5/6 SNX) with myocardial infarction (MI). Only a relatively small number of studies have employed such strategies—e.g., combining 5/6 nephrectomy (5/6 SNX) with MI—to mimic the multifactorial nature of CRS in humans (see also limitations). While the combination of 5/6 SNX with coronary artery ligation may represent a more clinically relevant preclinical approach for inducing concurrent cardiac and renal impairment in rodents, its robustness and translational value still require further confirmation in additional studies.

Similarly, inhibition of the nitric oxide pathway in spontaneously hypertensive rats (SHR) can offer a platform for studying end-stage renal disease by exacerbating systemic hypertension and accelerating renal injury. For example, in a study of the cardiovascular and renal protection afforded by an oral AT1-receptor blocker against L-NAME-exacerbated hypertension, chronic administration of L-NAME was used to suppress endogenous NO production in young SHRs [26]. More recently, Zhou et al. (2024) introduced a novel “two-hit” model of CRS based on inducing heart failure in SHRs by coronary artery ligation [254]. This model is in line with clinical observations, as in patients with pre-existing hypertension, myocardial infarction frequently precedes further deterioration in renal structure and function [254].

There is also a need to optimize the surgical procedures used in preclinical modeling of CRS, with the goals of improving survival, enhancing reproducibility, and reducing animal burden. Recent studies describe several refinements to the coronary artery ligation procedure, including improved surgical access to the heart while minimizing the risk of lung injury, continuous monitoring of body temperature and heart rate, and timely adjustment of retractors and anesthetic depth to prevent intraoperative cardiac complications. Implementation of these refinements has been shown to enhance scientific reliability and animal welfare by eliminating surgical mortality and achieving consistent myocardial infarction induction [279].

Another promising strategy involves the use of transcriptomic, proteomic, metabolomic, and non-coding RNA profiling to map convergent pathways in multifactorial conditions. Recent investigations employing both acute and chronic CRS models have identified previously unrecognized mediators of heart–kidney interactions [280]. As summarized in Table 1, several animal models exhibit increased expression of proteins associated with tissue remodeling and fibrosis (e.g., POSTN, TWEAK/Fn14, FGF23), immune-inflammatory activation (e.g., collagens, galectin-3, caveolins), RAAS hyperactivity (e.g., ACE), oxidative stress, and metabolic or mitochondrial dysregulation (e.g., CPT1, PTEN) in both the heart and kidneys [137,138,165,172,281,282,283]. Importantly, alterations in the cardiac and renal proteome can occur, even in the presence of ostensibly normal organ function. This may indicate that, for example, chronic heart failure may initiate a molecular injury program in the kidneys well before the development of overt uremia or clinically apparent renal dysfunction [280]. Among the many potential future directions for research, one may be to assess whether the gene-expression signatures identified in specific animal models faithfully replicate those observed in humans; while this would be a challenging objective, given the inherent heterogeneity of CRS, the findings would enhance the translational relevance of specific animal models. In addition, proteomic analyses may help identify early biomarkers and novel therapeutic targets for mitigating or preventing the progression of CRS.

In conclusion, identifying a rodent model that can mimic cardiorenal syndrome in patients remains a challenge. No animal approach comprehensively reflects the CRS model, according to the definition and classification for humans. Nevertheless Dahl/SS and ISO-HF replicate the renal consequences of heart injury for purposes of modeling CRS type 2, while DOCA-salt or STZ models were most likely to develop cardiac injury during progression of renal failure. Moreover, particular approaches provide valuable insights into the pathogenesis of this condition, be it inflammation (most models), oxidative stress (renal-ischemia–reperfusion, Dahl/SS, subtotal nephrectomy), neurohormonal disturbances (subtotal nephrectomy, uninephrectomized DOCA), and the role of systemic diseases (STZ-induced diabetic nephropathy, SHRs). Furthermore, from a practical point of view, such evaluations can be performed on an accelerated time frame (Table 2) [266,277].

To highlight temporal association and improve pathophysiological plausibility (clinical relevancy), the promising “two-hit” approaches could combine coronary artery ligation with 5/6SNX. In addition, to further enhance reproducibility, surgical procedures should be optimized to improve survival. 5/6SNX can accelerate cardiac changes post-MI whilst myocardial infarction can accelerate 5/6SNX-induced renal fibrosis, supporting bidirectional interactions in CRS. However, the value of the model needs to be confirmed by further research. This phenomenon also concerns hypertensive models. They can successfully replicate key features of human CRS; patients with pre-existing hypertension often experience myocardial infarction–induced deterioration of renal structure and function. For example, SHRs can develop cardiac dysfunction secondary to myocardial infarction followed by renal injury. The cardiorenal associations here could be explained by the activation of oxidative stress, inflammation and an imbalance in the RAAS—all features important in the pathogenesis of CRS.

### Strengths and Limitations

A key strength of this paper is that it is based on the results of a large number of experimental protocols (*n* = 251 papers). It also includes analyses based on subgroups according to the type of approach (animal model), and addresses the effects of changes in a variety of hemodynamic, echocardiographic, and histopathologic parameters related to heart and renal injury. These analyses were intended to reveal the potential of a particular model of one condition to provoke the occurrence of another. To reinforce their accuracy, the results of each comparison were only presented if the individual subgroup was included in at least three studies. Moreover this paper considers only studies where an individual model could develop both renal and cardiac injury, expressed by alterations in cardiac and renal parameters. Little publication bias was observed across the studies, and most of the included studies did not affect the pooled results with regard to individual parameters when subjected to leave-one-out analysis. Furthermore, data from each model was subjected to analysis based on the heterogeneity metric (Q) in most cases, the findings indicate an absence of internal variability (*p* > 0.05). In addition to the comparative analyses performed on animal model subgroups, meta-regression analyses were carried out to assess the potential correlation between particular cardiac and renal injury parameters.

The paper also has some limitations. Firstly, the papers were drawn from only two major databases (PubMed and Embase); however, these two sources provide comprehensive coverage of the literature relevant to this topic. Due to the limited number of experimental studies and comparisons, i.e., fewer than three, it was not possible to fully evaluate certain models based on the full spectrum of renal and cardiac parameters; these included “one-hit” models such as Goto-Kakizaki rat, SHR-SP, unilateral nephrectomy, 3/4 subtotal nephrectomy, Ren-2Tg, or doxorubicin-induced-HF, L-NAME-induced renal failure, and “two-hit” approaches such as Ren-2Tg+ACF or 5/6 SNX + doxorubicin-induced-HF.

In addition, due to the limited number of experimental studies performed on each model, and their considerable variety of renal and cardiac parameters, it was difficult to perform clear subgroup analyses using adequate sample sizes. Therefore, the analyses were restricted to the most frequently reported parameters and models. Hence, there is a need to develop more stringent and well-defined criteria for selecting and reporting data from experimental models. Such standardized criteria should specify renal and cardiac phenotypes that reflect the pathophysiological features observed in human CRS [284]. Possible renal criteria could include reductions in GFR, proteinuria and/or albuminuria, and renal fibrosis, whereas proposed cardiac parameters may encompass cardiac output, left ventricular hypertrophy, systolic (EF%) and/or diastolic dysfunction (LVEDP), hypertension, and myocardial fibrosis. These measures could be complemented by biomarker assessments, such as renal (e.g., cystatin C, kidney injury molecule-1, matrix metalloproteinase-9 or interleukin-18) and cardiac biomarkers (e.g., troponins, natriuretic peptides, homocysteine or C-reactive protein), to provide a better understanding of cardiorenal pathology.

The substantial heterogeneity observed between models may be partially attributed to various factors, such as animal age, strain and sex. Regarding animal strain, more pronounced renal and cardiovascular consequences were noted in 129/Sv mice than C57BL/6JRj mice. As most of the reviewed experiments were conducted using C57BL/6J mice, no further inter-strain comparative analysis was possible due to insufficient sample sizes. More definitive results were obtained for experimental duration (Supplementary Figure S2), and species (rat versus mouse, body weight parameter) (Supplementary Table S4).

## Figures and Tables

**Table 1:** Study characteristics.

Animal Model	Species	Median Age (Initial)	Description	Application	Experimental Period	Cardiac Features ^1,^*	Renal Features ^1,^*	Molecular Hallmarks
2K1C	R	9 wks.	Goldblatt two-kidney one-clip (2K1C) model of renovascular hypertension.	CRS-4	4–10 wks.	↑cardiac hypertrophy, ↑SBP	↑S-Cre	↑Ang II, ↑serum (urea) ACE activity, ↑BNP, ↑Il-6, ↑PAC, ↑TNF-alpha, ↑TGF-beta1
5/6SNX	M/R	8 wks.	Subtotal nephrectomy consisting of left partial nephrectomy (2/3), followed by a total right nephrectomy.	CRS-4	2–32 wks.	↑cardiac fibrosis, ↑cardiac hypertrophy, ↓EF, ↑LVEDP, ↑LVESP, ↑SBP	↑ACR, ↑BUN, ↑GI, ↓GFR, ↑kidney hypertrophy, ↑renal fibrosis, ↑S-Cre, ↑UAlbEx, ↑UProtEx	↑Ang II, ↑ANP, ↑BNP, ↑Ca, ↑cystatin C, ↑NT-proBNP, ↑FGF23, ↑Il-1beta, ↑Il-6, ↑MMP-2(9), ↑P, ↑PRA, ↓RBF, ↑TNF-alpha, ↑TGF-beta, ↑TnT, ↑urea, ↑UA, dysregulation of mitochondrial respiration in the heart
5/6SNX+MI (LAD-MI)	R	7.5 wks.	Subtotal nephrectomy with myocardial infarction.	CRS-2, CRS-4	2–12 wks.	↑cardiac fibrosis, ↑cardiac hypertrophy, ↑dP/dtmin, ↓EF, ↓FS, ↓LVESP, ↑LVEDP	↑ACR, ↑BUN, ↓GFR, ↑kidney hypertrophy, ↑renal fibrosis, ↑S-Cre, ↑UProtEx	
5/6SNX+ DOX-HF	R	No data	Subtotal nephrectomy with doxorubicin-induced heart failure.	CRS-2, CRS-4	2–8 wks.	↓EF, ↑LVESd	↑BUN, ↑S-Cre	
ACF	R	9 wks.	Aorto-caval fistula-induced heart failure.	CRS-2	1–21 wks.	↑cardiac hypertrophy, ↓EF, ↓FS, ↑LVEDd, ↑LVEDP, ↑LVESd	↓GFR, ↑UAlbEx	↑Ang II, ↑Ang 1–7, ↑BNP, ↑PAC, ↓RBF *ACE, RAGE, periostin, caveolin-1, collagen-VI, galectin-3*
ARF	M/R	8 wks.	Adenine-induced renal failure.	CRS-4	9–20 wks.	↑cardiac fibrosis, ↑cardiac hypertrophy	↑S-Cre	↑FGF23, ↑KIM-1, ↑MCP-1, ↑MPO, ↑NT-proBNP, ↑P-calc, ↑urea
bilateral R-IR	M/R	8 wks.	Acute kidney ischemia–reperfusion injury induced by a 15–45 min bilateral renal artery ischemia, followed by 24–72 h reperfusion.	CRS-3 CRS-4	1–7 days	↑cardiac hypertrophy, ↓EF	↑BUN, ↑S-Cre	↑Il-6, ↑TNF-alpha
Dahl/SS	R	6 wks.	A salt-sensitive rat strain genetically predisposed to elevated blood pressure when exposed to a high-salt diet (4–8% NaCl).	CRS-2	4–32 wks.	↑cardiac hypertrophy, ↓FS, ↑LVEDd, ↑LVEDP, ↑SBP	↑ACR, ↑BUN, ↓GFR, ↑GI, ↑kidney hypertrophy, ↑renal fibrosis, ↑S-Cre, ↑UAlbEx, ↑UProtEx	↑ANP, ↑BNP, ↑Ca, ↑E-selectin, ↑Il-1beta, ↑MCP-1, ↑NT-proBNP, ↑PAC, ↑PRA, ↑TGF-beta1, ↑LV NADPH oxidase *TRPC3, CSRP3*
DOX-HF	R	7 wks.	Doxorubicin-induced heart failure.	CRS-2	5–20 wks.		↑BUN, ↑S-Cre	
GK	R	11 wks.	Non-obese, non-hypertensive rat model of type 2 diabetes mellitus.	CRS-4	8–48 wks.		↑kidney hypertrophy	↑ANP *MYH7, MYL2, APOA1, phospholamban*
ISO-HF	R	6 wks.	Isoproterenol **-induced heart failure (cardiac injury, myocardial ischemia, hypertrophy, structural remodeling).	CRS-2	1–10 wks.	↑cardiac hypertrophy, ↓dP/dtmin, ↓dP/dtmax, ↓EF, ↓FS, ↑LVEDP	↑BUN, ↑kidney hypertrophy, ↑renal fibrosis	↑ Ang II, ↑NT-proBNP, ↑CK-MB, ↑cTnI, ↑MMP-9, ↑PAC, ↑renin
MI (LAD-MI)	M/R	8 wks.	Model of coronary artery ligation used to study myocardial infarction, ventricular remodeling, and congestive heart failure.	CRS-1, CRS-2	1 day–30 wks.	↑cardiac fibrosis, ↑cardiac hypertrophy, ↓EF, ↓FS, ↑LVEDP, ↑LVEDd, ↑LVESd, ↓LVESP, ↓SBP	↑BUN, ↓GFR, ↑renal fibrosis, ↑S-Cre, ↑UAlbEx, ↑UProtEx	↑BNP, ↑cystatin C, ↑Il-6, ↑Il-beta1, ↑NGAL, ↑urea *FGF23*
M-IR	M/R	12 wks.	Myocardial ischemia–reperfusion injury induced by a 30 min left coronary artery (LAD) ligation, followed by reperfusion.	CRS-1, CRS-2	1–28 days	↓EF	↑S-Cre	↑CK-MB, ↑cTnI, ↑Il-6, ↑MMP-9, ↑TNF-alpha *TWEAK-Fn14*
Ren-2Tg	R	9 wks.	The hypertensive Ren-2 transgenic rat (mREN2)27.	CRS-4	1–15 wks.	↑cardiac hypertrophy	↑GFR	↑Ang II
Ren-2Tg+ACF	R	9 wks.	The hypertensive Ren-2 transgenic rat (mREN2)27 exposed to aorto-caval fistula.	CRS-2	1–15 wks.	↑cardiac hypertrophy, ↓FS, ↑LVEDd, ↑LVESd		↑Ang II
SHR	R	9 wks.	Spontaneously hypertensive rat model of genetic hypertension and age-dependent left ventricular dysfunction.	CRS-2, mainly CRS-5	3–48 wks.	↑cardiac fibrosis, ↑cardiac hypertrophy, ↑SBP	↑kidney hypertrophy	
SHR-stroke prone	R	10 wks.	Spontaneously hypertensive stroke-prone rat model of non-surgery-induced stroke.	CRS-2, CRS-5	7–9 wks.	↑cardiac fibrosis, ↑cardiac hypertrophy, ↑SBP	↑UProtEx	↑PAC, ↑PRA
STZ	M/R	7 wks.	Streptozotocin-induced T1DM, T2DM (commonly combined with a high-fat diet), and diabetic nephropathy.	CRS-4, mainly CRS-5	4–20 wks.	↑cardiac fibrosis, ↑cardiac hypertrophy, ↓dP/dtmax, ↓dP/dtmin, ↓FS, ↑LVEDP	↑BUN, ↑kidney hypertrophy, ↑renal fibrosis, ↑S-Cre	↑Ang II, ↑BNP, ↑CK-MB, ↑cTnI, ↑TOC, ↑urea
TAC	M/R	8 wks.	Transverse aortic constriction used to induce pressure overload, resulting in heart failure.	CRS-2	1–18 wks.	↑cardiac hypertrophy, ↓EF, ↓FS, ↑LVEDd, ↑LVESd	↑ACR	↑BNP, ↑Il-1b, ↑MCP-1, ↑NA, ↑NT-proBNP, ↑renin, ↑TNF-alpha *PTEN, MTMR4, PLC, CPT1*
unilateral R-IR	M/R	7 wks.	Acute kidney ischemia–reperfusion injury induced by a 50–60 min unilateral renal artery ischemia, followed by reperfusion.	CRS-3, CRS-4	1–14 days	↑cardiac hypertrophy		↓eNOS, ↑Il-6, ↓SOD, ↑TNF-alpha, ↑urea
uninephrectomized DOCA	M/R	8 wks.	Uninephrectomy combined with DOCA administration and NACl induces chronic hypertension, which subsequently leads to cardiac hypertrophy.	CRS-4	2–11 wks.	↑cardiac fibrosis, ↑cardiac hypertrophy, ↑SBP	↑ACR, ↑GI, ↑kidney hypertrophy, ↑renal fibrosis, ↑S-Cre	↑Il-6, ↑KIM-1, ↑MCP-1, ↑NT-proBNP, ↑PAC, ↑PAI

^1^—At least three interventions must have been included into analysis; *—↓↑ demonstrate significant (*p* < 0.05) alterations in individual parameter according to results of analysis including at least three comparisons; **—across the included protocols, a wide range of ISO doses were employed (5–100 mg/kg); the most commonly used regimen was 5 mg/kg per day administered subcutaneously or intraperitoneally. Molecular circuits linking cardiac and renal injury, as identified through single-nucleus RNA sequencing (snRNA-seq) and proteomic analyses, were presented in *italics*. For detailed results, see Supplementary Table S4. 2K1C—2-kidney 1-clip; ACE—angiotensin converting enzyme; ACF—aorto-caval fistula; ACR—albumin to creatinine urinary excretion ratio; Ang II—angiotensin II; ANP—atrial natriuretic peptide; APOA1—apolipoprotein A-I; ARF—adenine-induced renal failure; BNP—B-type natriuretic peptide; BUN—blood urea nitrogen; Ca—serum calcium; CK-MB—creatine kinase MB; CPT1—carnitine palmitoyltransferase I; CSRP3—cysteine and glycine-rich protein 3; DOCA-salt—deoxycorticosterone acetate; Dox-HF—doxorubicin-induced heart failure; dP/dtmax—maximal rate of pressure increase; dP/dtmin—maximal rate of pressure decrease; EF—left ventricle ejection fraction; eNOS—endothelial nitric oxide synthase; FGF23—fibroblast growth factor 23; FS—fractional shortening; GI—glomerulosclerosis index; GFR—glomerular filtration rate; GK—Goto-Kakizaki; Il-1beta (Il-6)—interleukins; ISO-HF—isoproterenol-induced heart failure; KIM—kidney injury molecule; LAD-MI—left anterior descending coronary artery ligation; LV—left ventricle; LVEDd—left ventricular end-diastolic diameter; LVEDP—left ventricular end-diastolic pressure; LVESd—left ventricular entricle end-systolic diameter; LVESP—left ventricular end-systolic pressure; MCP-1—monocyte chemoattractant protein-1; MMP-2(9)—metaloproteins; MPO—myeloperoxidase; MTMR4—myotubularin-related protein 4; MYH7—myosin heavy chain 7; MYL2—myosin light chain 2; NA—noradrenaline; NGAL—neutrophil gelatinase-associated lipocalin; NT-proBNP—N-terminal pro-B-type natriuretic peptide; P-calc—calcium phosphorus; PAC—plasma aldosterone concentration; PAI—plasminogen activator inhibitor-1; PLC—phospholipase C; PRA—plasma renin activity; PTEN—phosphatase and tensin homolog; RBF—renal blood flow; Ren-2 Tg—(mRen2)27 transgenic; R-IR—renal ischemia–reperfusion; SBP—systolic blood pressure; S-Cre—serum creatinine; SHR—spontaneous hypertensive rat; SNX—subtotal nephrectomy; SOD—superoxide dismutase activity; STZ—streptozotocin injected; TAC—transverse aortic constriction; TGF-beta—transforming growth factor beta; TNF-alpha—tumor necrosis factor alpha; TnT—troponin T; TOC—total oxidative activity; TRPC3—transient receptor potential cation channel subfamily C member 3; TWEAK-Fn14—tumor necrosis factor-like weak inducer of apoptosis and its receptor fibroblast growth factor-inducible 14; UA—uric acid; UAlbEx—urinary albumin excretion; UNX—unilateral nephrectomy; UProtEx—urinary protein excretion.

**Table 2:** Take-home messages.

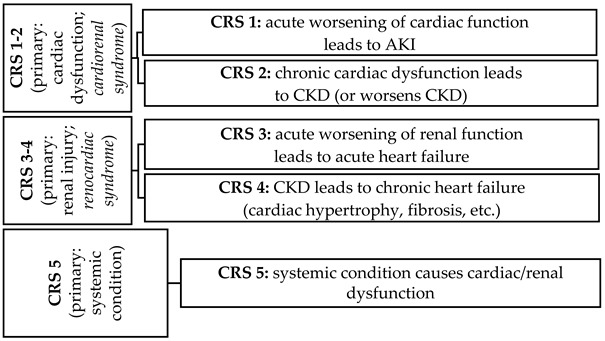
An animal model of cardiorenal syndrome should display the onset of one condition that temporally precedes the occurrence or progression of another condition
A wide spectrum of invasive and non-invasive animal approaches has been proposed to explore cardiorenal associations
Coronary artery ligation is one of the most widely assessed models for CRS types 1 and 2, and subtotal nephrectomy for chronic renocardiac syndrome (CRS-4)
In general, renal lesions were marked more significantly in CRS types 3 and 4, while available models of CRS types 1 and 2 developed numerous signs of more pronounced cardiac failure than models of renal injury (CRS-3 and 4).
In CRS type 2, “one-hit” models: Dahl/SS and ISO-HF demonstrated the most pronounced renal symptoms following heart injury, while DOCA-salt or STZ were the most effective at developing cardiac injury following renal failure
Myocardial hypertrophy was reported for most animal approaches with renal injury as the primary driver (CRS type 3 and 4). This phenomenon also involved animal models of ischemic or non-ischemic heart injury or isoproterenol-induced heart failure (CRS type 2)
In numerous approaches, cardiorenal impairments can be evoked by elevated systemic pressure. In CRS type 1 and 2, renal injury can result directly from elevated systolic pressure as well as from hypertension-mediated myocardial hypertrophy and heart failure. These models can successfully replicate key features of human CRS, as patients with pre-existing hypertension often experience deterioration of renal structure and function induced by myocardial infarction
The existing renocardiac models (CRS types 3 and 4) can manifest worse kidney function and renal fibrosis in response to a prolonged experimental period
Transcriptomic, proteomic, and non-coding RNA profiling represent promising approaches for mapping convergent pathways in CRS models, enabling the identification of previously unrecognized mediators of heart–kidney interactions.

## Data Availability

No new data were created or analyzed in this study. Data sharing is not applicable to this article.

## Supplementary Materials

The following supporting information can be downloaded at https://www.mdpi.com/article/10.3390/ijms27083477/s1.

## Abbreviations

The following abbreviations are used in this manuscript:

2K1C	2-kidney 1-clip
ACE	Angiotensin converting enzyme
ACF	Aorto-caval fistula
ACR	Albumin to creatinine urinary excretion ratio
AKI	Acute kidney injury
Ang II	Angiotensin II
ANP	Atrial natriuretic peptide
ARF	Adenine-induced renal failure
BNP	B-type natriuretic peptide
BUN	Blood urea nitrogen
BW	Body weight
Ca	Serum calcium
CKD	Chronic kidney disease
CK-MB	Creatine kinase MB
CoCl2-HF	Cobalt dichloride-induced HF
CRS	Cardiorenal syndrome
Dahl/SR	Dahl salt-resistant
Dahl/SS	Dahl salt-sensitive
DOCA-salt	Deoxycorticosterone acetate
double IR	Acute heart and kidney ischemia–reperfusion
Dox-HF	Doxorubicin-induced heart failure
dP/dtmax	Maximal rate of pressure increase
dP/dtmin	Maximal rate of pressure decrease
EF	Left ventricle ejection fraction
eNOS	endothelial nitric oxide synthase
FGF23	Fibroblast growth factor 23
FHH	Fawn-hooded hypertensive
FHL	Fawn-hooded low-pressure
FS	Fractional shortening
GFR	Glomerular filtration rate
GI	Glomerulosclerosis index
GK	Goto-Kakizaki
HanSD	Hannover Sprague-Dawley
HF	Heart failure
HFD	High-fat diet
HS	High salt
ISO-HF	Isoproterenol-induced heart failure
KIM	Kidney injury molecule
LAD-MI	Left anterior descending coronary artery ligation
L-NAME	N(ω)-nitro-L-arginine methyl ester
LS	Low salt
LV	Left ventricle
LVEDd	Left ventricular end-diastolic diameter
LVEDP	Left ventricular end-diastolic pressure
LVESd	Left ventricular end-systolic diameter
LVESP	Left ventricular end-systolic pressure
MCP-1	Monocyte Chemoattractant Protein-1
MI	Coronary artery ligation
M-IR	Myocardial ischemia–reperfusion
MMP-2(9)	Metaloproteins
MPO	Myeloperoxidase
NA	Noradrenaline
NGAL	Neutrophil gelatinase-associated lipocalin
NOS	Nitric oxide synthase
NT-proBNP	N-terminal pro-B-type natriuretic peptide
P	Calcium phosphorus
PAC	Pulmonary artery constriction
PAI	Plasminogen activator inhibitor-1
PRA	Plasma renin activity
RAAS	Renin–angiotensin–aldosterone system
RAL	Renal artery ligation
RBF	Renal blood flow
Ren-2 Tg	(mRen2)27 transgenic
R-IR	Renal ischemia–reperfusion
SBP	Systolic blood pressure
S-Cre	Serum creatinine
SD	Sprague-Dawley
SHR	Spontaneous hypertensive rat
SHR-SP	Stroke-prone spontaneously hypertensive rat
SNX	Subtotal nephrectomy
SOD	Superoxide dismutase activity
SR	Slow release
STZ	Streptozotocin
TAC	Transverse aortic constriction
TGF-beta	Transforming growth factor beta
TNF-alpha	Tumor necrosis factor alpha
TnT	Troponin T
TOC	Total oxidative activity
UA	Uric acid
UAlbEx	Urinary albumin excretion
UProtEx	Urinary protein excretion
UUO	Unilateral urinary obstruction
WD	Western diet
WKY	Wistar Kyoto
