# Supplementary material for: Cardiorenal Associations in Preclinical Modeling: A Systematic Review and Meta-Analysis
Source: Int J Mol Sci. 2026 Apr 13;27(8):3477. doi: 10.3390/ijms27083477 (PMC13116581; doi:10.3390/ijms27083477)
Supplement: Supplementary file 1 [file ijms-27-03477-s001.zip › ijms-4192546-supplementary.pdf]

Cardiorenal Associations in Preclinical Modeling: A Systematic Review and Meta-Analysis
Magdalena Jasińska-Stroschein

Supplementary. Search strategy

The databases (PUBMED and EMBASE; January 1992 to December 2024) were searched with no language restrictions using the following search terms in titles and abstracts:

(("heart failure"[MeSH Terms] OR ("heart"[All Fields] AND "failure"[All Fields]) OR "heart failure"[All Fields] OR ("cardiac"[All Fields] AND "failure"[All Fields]) OR "cardiac failure"[All Fields] OR ("heart failure"[MeSH Terms] OR ("heart"[All Fields] AND "failure"[All Fields]) OR "heart failure"[All Fields]) OR ("heart diseases"[MeSH Terms] OR ("heart"[All Fields] AND "diseases"[All Fields]) OR "heart diseases"[All Fields] OR ("heart"[All Fields] AND "disease"[All Fields]) OR "heart disease"[All Fields])) AND ("renal insufficiency"[MeSH Terms] OR ("renal"[All Fields] AND "insufficiency"[All Fields]) OR "renal insufficiency"[All Fields] OR ("renal"[All Fields] AND "failure"[All Fields]) OR "renal failure"[All Fields] OR ("kidney diseases"[MeSH Terms] OR ("kidney"[All Fields] AND "diseases"[All Fields]) OR "kidney diseases"[All Fields] OR ("kidney"[All Fields] AND "disease"[All Fields]) OR "kidney disease"[All Fields] OR cardiorenal syndrome)) AND ("mice"[MeSH Terms] OR "mice"[All Fields] OR "mouse"[All Fields] OR "mouse s"[All Fields] OR "mouses"[All Fields] OR ("rats"[MeSH Terms] OR "rats"[All Fields] OR "rat"[All Fields])))

Supplementary. Methods

The search criteria involved preclinical experiments on rodents (P = population) that were subjected to a variety of surgical or genetic interventions (I = intervention) to induce renal and cardiac lesions (O = outcome) in comparison to healthy subjects (C = comparator). Only preclinical experiments reporting the alterations in at least one "renal" parameter and at least one "cardiac" parameter were included in the analyses. The following were excluded: studies with missing data (e.g., the number of subjects or healthy animal data), *in vitro* studies, studies performed on isolated organs.

PubMed, and Embase were searched from January 1992 to December 2024 according to the search strategy. The following data were extracted: animals (species, race, sex, initial age), model featuring renal and cardiac impairments (surgical interventions, genetic modifications, diet, induction period), morphometric, laboratory, hemodynamic, echocardiographic and histopathologic parameters. For the purposes of quantitative analyses, the following data were extracted: morphometric parameters – body weight (BW), left ventricle (LV) mass, LV weight/tibia length (TL), LV/BW index, heart weight – HW/BW index, kidney mass, kidney mass/BW index; laboratory data – serum creatinine (S-Cre), blood urea nitrogen (BUN), urinary protein excretion ratio (UProtEx), urinary albumin excretion ratio (UAlbEx), urinary albumin to creatinine excretion ratio (ACR), glomerular filtration rate (GFR); hemodynamic data –systolic blood pressure (SBP), left ventricular systolic pressure (LVSP), left ventricular end-diastolic pressure (LVEDP), cardiac output (CO), cardiac index (CI), time needed for relaxation of 50% maximal left ventricular pressure to baseline (tau), maximal rate of pressure rise (dP/dtmax), maximal rate of pressure decline (dP/dtmin), echocardiographic data – left ventricular end-systolic diameter (LVESd), left ventricular end-diastolic diameter (LVEDd), left ventricular ejection fraction (EF), fractional shortening (FS), ratio of E-wave to A-wave (E/A); the histopathological data – myocardial fibrosis [interstitial (perivascular) fibrosis, fibrosis area or collagen fraction], glomerulosclerosis index score as well as

renal fibrosis. Other data concerned *inter alia* the fractional excretion of sodium (FENa%), renal blood floow (RBF), creatine kinase MB (CK-MB) and neutrophil gelatinase-associated lipocalin (NGAL).

The effect size, with a 95% confidence interval (CI), was expressed as difference in means (D) according to Equation (1): D = X(Model) − X(Sham), or was expressed as response ratio (R) according to Equation (2): R = X(Model) / X(Sham), where X – mean response in the group of animals; Sham – placebo control group (healthy subjects).

An increase in parameters LVEDP, LVESd, LVEDd, S-Cre, BUN, UProtEx, UAlbEx (Equation 1: D>0) or ACR, heart hypertrophy, renal hypertrophy, cardiac fibrosis, renal fibrosis, glomerulosclerosis index (Equation 2: R>1) and decrease in EF, FS, LVESP, dP/dtmax, dP/dtmin (Equation 1: D<0) or GFR (Equation 2: R<1) was assumed to indicate progression of cardio(renal) disease.

As pronounced heterogeneity was expected between studies, a random-effects model was chosen for analysis. The influence of model-related variables on the final outcome, subgroup analyses (qualitative variables e.g., animal species, race) or meta-regression (quantitative variables e.g., SBP, S-Cre, experimental period, UProtEx, UAlbEx) were performed, as pre-defined in the protocol.

Leave-one-out sensitivity analysis was used to assess the robustness of the results and to clarify the presence of individual comparisons that significantly affected the pooled results. Heterogeneity was assessed using Cochran's Q, with P≥0.05 suggesting the absence of heterogeneity.

The quality of each individual study was assessed using SYRCLE's risk of bias tool for animal studies. Publication bias between studies was evaluated using Egger's weighted regression with p≥0.05 suggesting the absence of missing studies and the Duval and Tweedie 'trim and fill' method. A p-value below 0.05 was considered statistically significant. The analyses were performed using STATISTICA 13.1 software.

Supplementary. S1 Table. Publication bias in relation to selected renal and cardiac parameters reported for particular animal models.

Parameter [unit] (effect size)	Animal model	Egger regression p-value	Imputed (trim and fill)
**ACR (R, response ratio)**	5/6SNX	=0.04	5
	5/6SNX+MI (LAD-MI)	=0.018	0
	Dahl/SS	NS	1
	MI (LAD-MI)	NS	3
	TAC	NS	0
	uninephrectomized DOCA	NS	2
**BUN [mg/dl] (D, difference in means)**	5/6SNX	=0.019	0
	5/6SNX+Dox-HF	NS	0
	5/6SNX+MI (LAD-MI)	NS	0
	bilateral R-IR	NS	0
	Dahl/SS	NS	0
	Dox-HF	NS	0
	ISO-HF	NS	0
	MI (LAD-MI)	NS	1
	STZ	NS	0
**Cardiac fibrosis (R, response ratio)**	5/6SNX	NS	3
	5/6SNX+MI (LAD-MI)	NS	0
	MI (LAD-MI)	NS	0
	SHR	NS	2
	SHR-stroke prone	NS	0
	STZ	NS	0
	uninephrectomized DOCA	NS	0
**dP/dt_{max} [mmHg/s] (D, difference in means)**	5/6SNX	NS	0
	5/6SNX+MI (LAD-MI)	NS	0
	ISO-HF	NS	0
	MI (LAD-MI)	=0.015	0
	STZ	NS	0
**dP/dt_{min} [mmHg/s] (D, difference in means)**	5/6SNX	NS	0
	5/6SNX+MI (LAD-MI)	NS	0
	ACF	NS	0
	ISO-HF	<0.0001	0
	MI (LAD-MI)	NS	0
	STZ	NS	0
**EF [%] (D, difference in means)**	5/6SNX	NS	0
	5/6SNX+Dox-HF	NS	0
	5/6SNX+MI (LAD-MI)	NS	2
	ACF	NS	0
	ARF	NS	0
	bilateral R-IR	NS	0
	Dahl/SS	NS	0
	ISO-HF	NS	0
	MI (LAD-MI)	NS	4
	M-IR	NS	0
	TAC	NS	0
	uninephrectomized DOCA	NS	0
**FS [%] (D, difference in means)**	5/6SNX	NS	4
	5/6SNX+MI (LAD-MI)	NS	0
	ACF	NS	0
	bilateral R-IR	NS	0
	Dahl/SS	NS	0
	ISO-HF	NS	0
	MI (LAD-MI)	=0.001	3
	Ren-2Tg+ACF	=0.017	0
	STZ	NS	0
	TAC	NS	0
	uninephrectomized DOCA	NS	0
	5/6SNX	NS	0

**GFR (R, response ratio)**	5/6SNX+MI (LAD-MI)	NS	0
	ACF	NS	0
	bilateral R-IR	=0.015	0
	Dahl/SS	NS	0
	MI (LAD-MI)	NS	0
	Ren-2Tg	NS	0
	Ren-2Tg+ACF	NS	0
**Glomerulosclerosis index score (R, response ratio)**	5/6SNX	NS	0
	Dahl/SS	NS	2
	uninephrectomized DOCA	NS	0
**Kidney hypertrophy (R, response ratio)**	5/6SNX	NS	0
	5/6SNX+MI (LAD-MI)	NS	0
	ACF	NS	0
	Dahl/SS	NS	0
	Goto-Kakizaki	NS	0
	ISO-HF	NS	0
	MI (LAD-MI)	NS	1
	Ren-2Tg	NS	0
	Ren-2Tg+ACF	NS	0
	SHR	NS	0
	SHR-stroke prone	NS	0
	STZ	NS	0
	TAC	NS	0
	uninephrectomized DOCA	NS	0
**LVEDd [mm] (D, difference in means)**	5/6SNX	NS	3
	5/6SNX+Dox-HF	NS	0
	5/6SNX+MI (LAD-MI)	NS	0
	ACF	NS	0
	Dahl/SS	NS	0
	MI (LAD-MI)	NS	0
	Ren-2Tg	NS	0
	Ren-2Tg+ACF	NS	0
	TAC	NS	0
**LVEDP [mmHg] (D, difference in means)**	5/6SNX	NS	0
	5/6SNX+MI (LAD-MI)	NS	0
	ACF	NS	0
	bilateral R-IR	=0.04	0
	Dahl/SS	NS	0
	ISO-HF	NS	0
	MI (LAD-MI)	=0.0003	2
	STZ	NS	0
	uninephrectomized DOCA	NS	0
	uninephrectomy+MI (LAD-MI)	NS	0
**LVESd [mm] (D, difference in means)**	5/6SNX	NS	0
	5/6SNX+MI (LAD-MI)	NS	0
	5/6SNX+Dox-HF	NS	0
	ACF	NS	0
	Dahl/SS	NS	0
	MI (LAD-MI)	NS	1
	Ren-2Tg	NS	0
	Ren-2Tg+ACF	NS	0
	TAC	NS	0
**LVESP [mmHg] (D, difference in means)**	5/6SNX	NS	0
	ISO-HF	NS	0
	MI (LAD-MI)	NS	0
**Myocardial hypertrophy (R, response ratio)**	2K1C	NS	0
	5/6SNX	=0.046	1
	5/6SNX+MI (LAD-MI)	NS	0
	ACF	NS	0
	ARF	NS	0
	bilateral R-IR	NS	0

	Dahl/SS	NS	0
	ISO-HF	NS	0
	MI (LAD-MI)	=0.003	7
	Ren-2Tg	NS	0
	Ren-2Tg+ACF	=0.016	0
	SHR	NS	0
	SHR-stroke prone	NS	1
	STZ	=0.01	1
	TAC	NS	0
	unilateral R-IR	=0.026	0
	uninephrectomized DOCA	NS	0
	UNX+ACF	NS	0
**SBP [mmHg] (D, difference in means)**	2K1C	NS	0
	5/6SNX	NS	9
	5/6SNX+MI (LAD-MI)	NS	0
	ARF	NS	0
	Dahl/SS	NS	2
	MI (LAD-MI)	NS	0
	SHR	NS	0
	SHR-stroke prone	NS	0
	STZ	NS	0
	TAC	NS	0
	uninephrectomized DOCA	NS	0
**S-Cre [mg/dl] (D, difference in means)**	2K1C	NS	0
	5/6SNX	<0.0001	11
	5/6SNX+MI (LAD-MI)	NS	0
	5/6SNX+Dox-HF	NS	0
	ACF	NS	0
	ARF	NS	0
	bilateral R-IR	NS	0
	Dahl/SS	NS	0
	Dox-HF	NS	0
	ISO-HF	NS	0
	MI (LAD-MI)	<0.0001	9
	M-IR	NS	0
	STZ	NS	1
	TAC	=0.007	0
	unilateral R-IR	NS	0
	uninephrectomized DOCA	NS	0
**Renal fibrosis (R, response ratio)**	5/6SNX	=0.032	0
	5/6SNX+(LAD)MI	NS	0
	Dahl/SS	NS	0
	ISO-HF	NS	1
	MI (LAD-MI)	0.013	0
	STZ	NS	0
	uninephrectomized DOCA	NS	0
**UAlbEx [mg/24h] (D, difference in means)**	5/6SNX	=0.03	8
	ACF	NS	1
	Dahl/SS	NS	0
	MI (LAD-MI)	NS	0
	STZ	NS	0
**UProtEx [mg/24h] (D, difference in means)**	5/6SNX	=0.012	6
	5/6SNX+MI (LAD-MI)	NS	3
	Dahl/SS	NS	0
	MI (LAD-MI)	NS	2
	SHR-stroke prone	NS	0
	UNX+MI	NS	0
**BW [g] (D, difference in means)**	2K1C	NS	0
	5/6SNX	=0.004	0
	5/6SNX+MI (LAD-MI)	NS	0
	ACF	NS	0
	ARF	=0.05	0
	Dahl/SS	NS	0
	ISO-HF	NS	0
	MI (LAD-MI)	NS	0

	Ren-2Tg	NS	0
	Ren-2Tg+ACF	NS	0
	SHR	NS	0
	SHR-stroke prone	NS	0
	STZ	NS	0
	TAC	=0.06	0
	unilateral R-IR	NS	0
	uninephrectomized DOCA	=0.04	0

Non-significant Egger regression value and lack of missing studies (trim and fill procedure) indicate low possibility of publication bias. 2K1C – 2-kidney 1-clip; ACF – aorto-caval fistula; ACR – albumin to creatinine urinary excretion ratio; ARF – adenine-induced renal failure; BUN – blood urea nitrogen; BW – body weight; DOCA-salt – deoxycorticosterone acetate; Dox-HF – doxorubicin-induced heart failure; dP/dt_{max} – maximal rate of pressure increase; dP/dt_{min} – maximal rate of pressure decrease; EF – left ventricle ejection fraction; FS – fractional shortening; GFR – glomerular filtration rate; GK – Goto-Kakizaki; ISO-HF – isoproterenol-induced HF; LAD-MI – left anterior descending coronary artery ligation; LVEDd – left ventricular end-diastolic diameter; LVEDP – left ventricular end-diastolic pressure; LVESd – left ventricular entricle end-systolic diameter; LVESP – left ventricular end-systolic pressure; Ren-2 Tg – (mRen2)27 transgenic; R-IR – renal ischemia-reperfusion; SBP – systolic blood pressure; S-Cre – serum creatinine; SHR – spontaneous hypertensive rat; SNX – subtotal nephrectomy; STZ – streptozotocin injected; TAC – transverse aortic constriction; UAlbEx – urinary albumin excretion; UNX – unilateral nephrectomy; UProtEx – urinary protein excretion; UUO – unilateral urinary obstruction.

Supplementary. S2 Table. Sensitivity analysis.

**Parameter [unit] (effect size)**	**Animal model**	**Item**	**Mean**	**Stand. error**	**p-value**	**Change in standard error***	**No of trials /interventions****
**ACR (R, response ratio)**	5/6SNX	**Overall effect**	**3.36**	**0.6**	**<0.0001**	-	**10**
		Statistics with study removed	4.42	0.99	<0.0001	64.52%	
			2.04	0.23	<0.0001	-61.06%	
	5/6SNX+MI (LAD-MI)	**Overall effect**	**2.10**	**0.42**	**=0.0002**	-	**4**
		Statistics with study removed	1.61	0.14	<0.0001	-67.00%	
	Dahl/SS	**Overall effect**	**7.46**	**1.49**	**<0.0001**	-	**4**
		Statistics with study removed	9.58	1.88	<0.0001	26.17%	
			6.08	0.73	<0.0001	-51.09%	
	MI (LAD-MI)	**Overall effect**	**1.26**	**0.18**	**NS**	-	**10**
		Statistics with study removed	1.31	0.23	NS	30.98%	
			1.09	0.13	NS	-24.36%	
	TAC	**Overall effect**	**2.36**	**0.40**	**<0.0001**	-	**5**
		Statistics with study removed	2.44	0.75	<0.0001	87.04%	
			2.03	0.30	<0.0001	-24.75%	
	uninephrectomized DOCA	**Overall effect**	**3.47**	**0.51**	**<0.0001**	-	**5**
		Statistics with study removed	3.47	0.53	<0.0001	3.47%	
			3.29	0.30	<0.0001	-41.32%	
**BUN [mg/dl] (D, difference in means)**	5/6SNX	**Overall effect**	**41.92**	**2.67**	**<0.0001**	-	**36**
		Statistics with study removed	42.59	2.88	<0.0001	7.96%	
			41.69	2.56	<0.0001	-4.09%	
	5/6SNX+Dox-HF	**Overall effect**	**67.16**	**21.44**	**=0.0017**	-	**3**
		Statistics with study removed	45.97	22.00	=0.037	2.62%	
			88.83	21.00	<0.0001	-2.04%	
	5/6SNX+MI (LAD-MI)	**Overall effect**	**49.71**	**14.48**	**=0.0006**	-	**3**
		Statistics with study removed	45.64	20.40	=0.02	40.87%	
			62.92	3.89	<0.0001	-73.12%	
	bilateral R-IR	**Overall effect**	**111.93**	**11.73**	**<0.0001**	-	**10**
		Statistics with study removed	111.05	13.36	<0.0001	13.92%	
			118.93	9.75	<0.0001	-16.82%	
	Dahl/SS	**Overall effect**	**4.80**	**1.93**	**=0.012**	-	**6**
		Statistics with study removed	1.92	0.71	=0.006	-63.41%	
			5.53	2.74	=0.04	42.45%	
	Dox-HF	**Overall effect**	**58.29**	**12.81**	**<0.0001**	-	**3**
		Statistics with study removed	40.54	13.50	=0.003	5.93%	
	ISO-HF	**Overall effect**	**12.47**	**6.19**	**=0.044**	-	**3**

		Statistics with study removed	6.03	1.02	<0.0001	-83.58%	
	MI (LAD-MI)	**Overall effect**	**5.99**	**1.60**	**=0.0002**	-	**13**
		Statistics with study removed	6.91	2.32	=0.003	44.82%	
			2.99	1.39	=0.03	-13.30%	
	STZ	**Overall effect**	**23.63**	**5.51**	**<0.0001**	**-**	**5**
		Statistics with study removed	26.23	8.83	=0.003	60.07%	
			15.75	5.25	=0.003	-4.86%	
**Cardiac fibrosis (R. response ratio)**	5/6SNX	**Overall effect**	**2.43**	**0.34**	**<0.0001**	-	**19**
		Statistics with study removed	2.46	0.41	<0.0001	20.08%	
			2.23	0.32	<0.0001	-6.48%	
	5/6SNX+MI (LAD-MI)	**Overall effect**	**2.28**	**0.96**	**NS**	-	**3**
		Statistics with study removed	2.39	1.22	NS	26.16%	
			1.84	0.88	NS	-8.50%	
	MI (LAD-MI)	**Overall effect**	**2.41**	**0.46**	**<0.0001**	-	**7**
		Statistics with study removed	2.74	0.80	=0.0006	73.68	
			1.95	0.34	=0.0001	-25.11%	
	SHR	**Overall effect**	**3.00**	**0.24**	**<0.0001**	**-**	**3**
		Statistics with study removed	3.00	0.35	<0.0001	42.49%	
	SHR-stroke prone	**Overall effect**	**2.62**	**0.81**	**=0.002**	**-**	**3**
		Statistics with study removed	3.45	1.56	=0.006	92.65%	
			2.22	0.52	=0.0007	-35.82%	
	STZ	**Overall effect**	**3.60**	**0.65**	**<0.0001**	-	**6**
		Statistics with study removed	3.60	0.88	<0.0001	35.32%	
			2.94	0.45	<0.0001	-30.61%	
	uninephrectomized DOCA	**Overall effect**	**4.07**	**1.01**	**<0.0001**	**-**	**8**
		Statistics with study removed	4.56	1.35	<0.0001	34.11%	
			3.54	0.71	<0.0001	-29.41%	
**dP/dt_{max} [mmHg/s] (D, difference in means)**	5/6SNX	**Overall effect**	**-1190.75**	**778.35**	**NS**	-	**7**
		Statistics with study removed	-1203.83	1231.15	NS	58.17%	
			-485.33	668.96	NS	-14.05%	
	ISO-HF	**Overall effect**	**-4840.85**	**1061.94**	**<0.0001**	-	**4**
		Statistics with study removed	-4604.74	1237.79	=0.0002	16.56%	
			-5673.86	199.73	<0.0001	-81.19%	
	MI (LAD-MI)	**Overall effect**	**-2218.43**	**292.04**	**<0.0001**	-	**18**
		Statistics with study removed	-2299.12	317.04	<0.0001	8.56%	
			-1897.58	260.57	<0.0001	-10.77%	
	STZ	**Overall effect**	**-1735.47**	**426.47**	**<0.0001**	**-**	**4**
		Statistics with study removed	-2069.70	622.97	<0.0001	46.07%	
**dP/dt_{min} [mmHg/s] (D, difference in means)**	5/6SNX	**Overall effect**	**-2310.32**	**669.56**	**=0.0006**	-	**5**
		Statistics with study removed	-2598.08	1015.37	=0.015	51.65%	
			-1661.61	529.41	=0.0017	-20.93%	
	5/6SNX+MI (LAD-MI)	**Overall effect**	**-2950.62**	**983.89**	**=0.0027**	**-**	**3**
		Statistics with study removed	-1522.44	336.69	<0.0001	-65.78%	
	ISO-HF	**Overall effect**	**-3967.19**	**1247.40**	**<0.0001**	**-**	**3**
		Statistics with study removed	-3449.01	1544.40	<0.0001	23.81%	
			-5037.00	376.26	<0.0001	-69.84%	
	MI (LAD-MI)	**Overall effect**	**-1896.00**	**172.56**	**<0.0001**	**-**	**14**
		Statistics with study removed	-1914.92	205.40	<0.0001	19.03%	
			-1792.81	163.92	<0.0001	-5.01%	
	STZ	**Overall effect**	**-1192.17**	**227.10**	**<0.0001**	-	**4**
		Statistics with study removed	-1597.64	349.25	<0.0001	53.79%	
			-627.86	125.96	<0.0001	-44.54%	
**EF [%] (D, difference in means)**	5/6SNX	**Overall effect**	**-5.56**	**2.03**	**=0.006**	**-**	**28**
		Statistics with study removed	-5.69	2.22	=0.01	9.17%	
			-4.35	1.86	=0.019	-8.36%	
	5/6SNX+Dox-HF	**Overall effect**	**-15.66**	**4.43**	**=0.0004**	-	**6**
		Statistics with study removed	-16.79	5.39	=0.0018	21.77%	
			-12.78	3.97	=0.0013	-10.31%	
	5/6SNX+MI (LAD-MI)	**Overall effect**	**-40.65**	**3.47**	**<0.0001**	-	**9**
		Statistics with study removed	-40.11	4.01	<0.0001	15.82%	
			-42.65	3.27	<0.0001	-5.64%	

	ACF	**Overall effect**	**-12.13**	**5.18**	**=0.019**	-	**3**
		Statistics with study removed	-17.21	3.03	<0.0001	-41.49%	
	ARF	**Overall effect**	**-3.48**	**5.25**	**NS**	**-**	**3**
		**Statistics with study removed**	**-1.23**	**7.83**	**NS**	**49.14%**	
			**-7.73**	**0.64**	**P<0.0001**	**-87.19%**	
	bilateral R-IR	**Overall effect**	**-5.88**	**2.44**	**=0.016**	-	**3**
		Statistics with study removed	-6.38	3.06	=0.037	25.15%	
			-3.72	0.92	=0.0001	-62.35%	
	Dahl/SS	**Overall effect**	**-2.66**	**2.27**	**NS**	-	**7**
		Statistics with study removed	-3.31	3.27	NS	44.41%	
			-0.47	1.92	NS	-15.42%	
	ISO-HF	**Overall effect**	**-23.74**	**5.15**	**<0.0001**	**-**	**6**
		Statistics with study removed	-24.28	6.37	<0.0001	23.72%	
			-19.30	4.00	<0.0001	-22.24%	
	MI (LAD-MI)	**Overall effect**	**-28.44**	**4.28**	**<0.0001**	-	**27**
		Statistics with study removed	-28.34	5.08	<0.0001	18.51%	
			-31.19	3.53	<0.0001	-17.65%	
	M-IR	**Overall effect**	-21.79	4.50	**<0.0001**		**4**
		Statistics with study removed	-21.17	7.27	=0.004	61.59%	
			-26.32	1.20	<0.0001	-73.41%	
	TAC	**Overall effect**	**-19.67**	**5.59**	**=0.0004**	**-**	**12**
		Statistics with study removed	-19.36	6.08	=0.0014	8.72%	
			-17.60	5.06	=0.0005	-9.49%	
	uninephrectomized DOCA	**Overall effect**	**0.66**	**3.74**	**NS**	**-**	**6**
		Statistics with study removed	1.20	4.30	NS	15.06%	
			-1.61	1.43	NS	-61.65%	
**FS [%] (D, difference in means)**	5/6SNX	**Overall effect**	**-1.85**	**1.90**	**NS**	**-**	**21**
		Statistics with study removed	-1.57	2.06	NS	8.70%	
			-2.88	1.89	NS	-0.61%	
	5/6SNX+MI (LAD-MI)	**Overall effect**	**-25.17**	**2.49**	**<0.0001**	**-**	**8**
		Statistics with study removed	-25.32	3.08	<0.0001	23.33%	
			-23.39	1.08	<0.0001	-56.86%	
	ACF	**Overall effect**	**-18.72**	**1.18**	**<0.0001**	**-**	**4**
		Statistics with study removed	-19.57	1.75	<0.0001	48.09%	
	bilateral R-IR	**Overall effect**	**-0.61**	**2.20**	**NS**	**-**	**10**
		Statistics with study removed	-0.93	2.43	NS	10.52%	
			0.77	1.91	NS	-13.28%	
	Dahl/SS	**Overall effect**	**-13.51**	**4.01**	**=0.0007**	-	**10**
		Statistics with study removed	-13.14	5.43	=0.0155	35.54%	
			-14.75	3.99	=0.0002	-0.45%	
	ISO-HF	**Overall effect**	**-19.23**	**6.97**	**=0.006**	**-**	**6**
		Statistics with study removed	-19.07	8.90	=0.032	27.75%	
			-13.27	2.35	<0.0001	-66.27%	
	MI (LAD-MI)	**Overall effect**	**-25.10**	**0.45**	**<0.0001**	**-**	**27**
		Statistics with study removed	-24.50	0.53	<0.0001	18.97%	
			-24.82	0.44	<0.0001	-1.65%	
	Ren-2Tg+ACF	**Overall effect**	**-15.24**	**3.59**	**<0.0001**	-	**3**
		Statistics with study removed	-13.89	5.05	=0.006	40.68%	
			-18.47	0.94	<0.0001	-73.89%	
	STZ	**Overall effect**	**-16.06**	**3.71**	**<0.0001**	**-**	**4**
		Statistics with study removed	-15.18	5.64	=0.014	52.00%	
			-21.29	1.71	<0.0001	-53.93%	
	TAC	**Overall effect**	**-18.35**	**3.16**	**<0.0001**	**-**	**6**
		Statistics with study removed	-17.72	4.07	<0.0001	28.87%	
			-16.08	2.59	<0.0001	-17.95%	
	uninephrectomized DOCA	**Overall effect**	**-0.23**	**1.53**	**NS**	**-**	**3**
		Statistics with study removed	-1.01	2.00	NS	31.31%	
			0.88	1.34	NS	12.18%	
	5/6SNX	**Overall effect**	**0.41**	**0.12**	**=0.003**	**-**	**9**
		Statistics with study removed	0.41	0.16	=0.02	27.10%	
			0.34	0.10	=0.0001	-21.49%	

	5/6SNX+MI (LAD-MI)	**Overall effect**	**0.28**	**0.14**	**=0.012**	**-**	**4**
		Statistics with study removed	0.34	0.16	=0.02	11.04%	
			0.18	0.08	=0.0003	-40.32%	
	ACF	**Overall effect**	**0.74**	**0.09**	**=0.018**	**-**	**7**
		Statistics with study removed	0.75	0.10	=0.038	10.83%	
			0.65	0.05	=0.0001	-42.54%	
	bilateral R-IR	**Overall effect**	**0.08**	**0.11**	**NS**	**-**	**4**
		Statistics with study removed	0.11	0.18	NS	68.04%	
**GFR (R, response ratio)**	Dahl/SS	**Overall effect**	**0.64**	**0.11**	**=0.01**	**-**	**4**
		Statistics with study removed	0.57	0.11	=0.005	1.89%	
			0.57	0.11	=0.004	1.06%	
	MI (LAD-MI)	**Overall effect**	**0.77**	**0.05**	**<0.0001**	**-**	**14**
		Statistics with study removed	0.77	0.06	<0.0001	25.62%	
			0.80	0.04	<0.0001	-5.33%	
	Ren-2Tg	**Overall effect**	**1.16**	**0.03**	**<0.0001**	**-**	**3**
		Statistics with study removed	1.16	0.06	=0.005	75.96%	
	Ren-2Tg+ACF	**Overall effect**	**0.96**	**0.08**	**NS**	**-**	**3**
		Statistics with study removed	0.94	0.15	NS	94.12%	
			1.03	0.06	NS	-19.90%	
**Glomerulosclerosis index score (R, response ratio)**	5/6SNX	**Overall effect**	**2.49**	**0.38**	**<0.0001**	**-**	**12**
		Statistics with study removed	2.63	0.45	<0.0001	18.24%	
			2.34	0.31	<0.0001	-19.60%	
	Dahl/SS	**Overall effect**	**8.30**	**1.52**	**<0.0001**	**-**	**5**
		Statistics with study removed	9.90	1.91	<0.0001	25.69%	
			7.26	1.34	<0.0001	-12.32%	
	MI (LAD-MI)	**Overall effect**	**2.19**	**0.83**	**=0.037**	**-**	**3**
		Statistics with study removed	**2.53**	**2.26**	**NS**	**172.86%**	
	uninephrectomized DOCA	**Overall effect**	**5.48**	**2.10**	**<0.0001**	**-**	**5**
		Statistics with study removed	7.93	2.84	<0.0001	34.77%	
			4.65	1.87	<0.0001	-11.13%	
	5/6SNX	**Overall effect**	**1.34**	**0.12**	**=0.0009**	**-**	**8**
		Statistics with study removed	1.33	0.15	=0.01	25.14%	
			1.44	0.07	<0.0001	41.32%	
	5/6SNX+MI (LAD-MI)	**Overall effect**	**1.46**	**0.07**	**<0.0001**	**-**	**3**
		Statistics with study removed	1.56	0.04	<0.0001	-42.79%	
	ACF	**Overall effect**	**1.01**	**0.03**	**NS**	**-**	**7**
		Statistics with study removed	1.02	0.05	NS	49.25%	
			0.97	0.03	NS	25.15%	
	Dahl/SS	**Overall effect**	**1.63**	**0.26**	**=0.002**	**-**	**9**
		Statistics with study removed	1.68	0.32	=0.007	25.27%	
			1.79	0.15	<0.0001	-43.54%	
	Goto-Kakizaki	**Overall effect**	**1.23**	**0.02**	**<0.0001**	-	**3**
**Kidney hypertrophy (R, response ratio)**		Statistics with study removed	1.24	0.03	<0.0001	69.66%	
	ISO-HF	**Overall effect**	**1.12**	**0.04**	**=0.002**	**-**	**4**
		Statistics with study removed	1.16	0.01	<0.0001	-70.86%	
	MI (LAD-MI)	**Overall effect**	**1.03**	**0.02**	**NS**	**-**	**16**
		Statistics with study removed	1.02	0.02	NS	-3.89%	
			1.03	0.02	NS	15.83%	
	Ren-2Tg	**Overall effect**	**1.06**	**0.05**	**NS**	**-**	**6**
		Statistics with study removed	1.05	0.07	NS	37.14%	
			1.02	0.02	NS	-56.32%	
	Ren-2Tg+ACF	**Overall effect**	**1.01**	**0.01**	**NS**	**-**	**6**
		Statistics with study removed	0.99	0.02	NS	56.60%	
	SHR	**Overall effect**	**1.05**	**0.01**	**<0.0001**	**-**	**3**
		Statistics with study removed	1.06	0.01	<0.0001	26.75%	
			1.05	0.01	<0.0001	-24.83%	
	SHR-stroke prone	**Overall effect**	**1.57**	**0.37**	**NS**	**-**	**3**
			1.57	0.48	NS	28.81%	

		Statistics with study removed	1.31	0.19	NS	-48.69%	
	STZ	**Overall effect**	**1.70**	**0.16**	**<0.0001**	**-**	**7**
		Statistics with study removed	1.69	0.19	<0.0001	18.87%	
			1.83	0.15	<0.0001	-5.50%	
	TAC	**Overall effect**	**0.98**	**0.08**	**NS**	**-**	**3**
		Statistics with study removed	0.97	0.10	NS	33.60%	
			1.06	0.04	NS	-45.14%	
	uninephrectomized DOCA	**Overall effect**	**2.15**	**0.18**	**<0.0001**	**-**	**9**
		Statistics with study removed	2.05	0.06	<0.0001	-64.00%	
			2.15	0.21	<0.0001	15.89%	
**LVEDd [mm] (D, difference in means)**	5/6SNX	**Overall effect**	**0.36**	**0.19**	**NS**	**-**	**18**
		Statistics with study removed	0.38	0.22	NS	14.25%	
			0.27	0.16	NS	-15.97%	
	5/6SNX+Dox-HF	**Overall effect**	**0.19**	**0.19**	**NS**	**-**	**3**
		Statistics with study removed	0.24	0.35	NS	82.13%	
			0.05	0.11	NS	-41.13%	
	5/6SNX+MI (LAD-MI)	**Overall effect**	**1.71**	**1.05**	**NS**	**-**	**3**
		Statistics with study removed	2.00	1.49	NS	42.35%	
	ACF	**Overall effect**	**4.23**	**0.85**	**<0.0001**	**-**	**6**
		Statistics with study removed	4.66	1.31	<0.0001	53.95%	
			3.72	0.78	<0.0001	-8.62%	
	Dahl/SS	**Overall effect**	**0.64**	**0.32**	**=0.047**	**-**	**9**
		Statistics with study removed	0.74	0.38	=0.048	17.29%	
			0.85	0.30	=0.005	-6.03%	
	MI (LAD-MI)	**Overall effect**	**1.73**	**0.20**	**<0.0001**	**-**	**18**
		Statistics with study removed	1.72	0.22	<0.0001	11.60%	
			1.84	0.18	<0.0001	-9.49%	
	Ren-2Tg	**Overall effect**	**-0.93**	**0.48**	**=0.05**	**-**	**3**
		Statistics with study removed	-0.23	0.29	NS	-39.49%	
	Ren-2Tg+ACF	**Overall effect**	**4.03**	**0.41**	**<0.0001**	**-**	**3**
		Statistics with study removed	4.38	0.17	<0.0001	-58.84%	
			3.87	0.57	<0.0001	39.10%	
	TAC	**Overall effect**	**0.41**	**0.20**	**=0.04**	**-**	**7**
		Statistics with study removed	0.50	0.24	=0.03	18.50%	
			0.17	0.08	=0.03	-61.78%	
**LVEDP [mmHg] (D, difference in means)**	5/6SNX	**Overall effect**	**3.67**	**0.57**	**<0.0001**	**-**	**10**
		Statistics with study removed	3.79	0.68	<0.0001	18.07%	
			3.32	0.56	<0.0001	-2.67%	
	5/6SNX+MI (LAD-MI)	**Overall effect**	**5.70**	**0.84**	**<0.0001**	**-**	**3**
		Statistics with study removed	5.48	1.30	<0.0001	55.05%	
			6.52	0.48	<0.0001	-42.50%	
	ACF	**Overall effect**	**5.63**	**1.05**	**<0.0001**	**-**	**3**
		Statistics with study removed	5.55	1.37	<0.0001	30.17%	
			6.76	0.73	<0.0001	-30.58%	
	bilateral R-IR	**Overall effect**	**0.38**	**0.31**	**NS**	**-**	**4**
		Statistics with study removed	0.79	0.52	NS	67.78%	
	Dahl/SS	**Overall effect**	**8.21**	**3.30**	**=0.01**	**-**	**3**
		Statistics with study removed	11.24	1.98	<0.0001	-40.00%	
	ISO-HF	**Overall effect**	**12.75**	**0.92**	**<0.0001**	**-**	**4**
		Statistics with study removed	12.87	1.01	<0.0001	10.00%	
			13.81	0.53	<0.0001	-42.78%	
	MI (LAD-MI)	**Overall effect**	**11.51**	**1.60**	**<0.0001**	**-**	**20**
		Statistics with study removed	11.86	1.65	<0.0001	2.73%	
			11.26	1.51	<0.0001	-5.96%	
	STZ	**Overall effect**	**4.83**	**1.92**	**=0.01**	**-**	**3**
		Statistics with study removed	6.58	2.58	=0.018	34.26%	
			2.74	1.32	=0.038	-31.30%	
	Uninephrecto-mized DOCA	**Overall effect**	**5.59**	**3.00**	**=0.06**	**-**	**3**
		Statistics with study removed	4.44	3.70	NS	23.21%	
			8.13	0.75	<0.0001	-74.93%	

	Uninephrecto-my + MI (LAD-MI)	**Overall effect**	**5.02**	**2.20**	**=0.02**	**-**	**3**
		**Statistics with study removed**	**6.80**	**5.04**	**NS**	**129.74%**	
			**1.8**	**0.28**	**<0.0001**	**-87.16%**	
**LVESd [mm] (D, difference in means)**	5/6SNX	**Overall effect**	**-0.26**	**0.12**	**=0.035**	**-**	**12**
		**Statistics with study removed**	**-0.27**	**0.14**	**NS**	**18.89%**	
			**-0.17**	**0.10**	**NS**	**-14.99%**	
	5/6SNX+MI (LAD-MI)	**Overall effect**	**2.76**	**1.27**	**=0.029**	**-**	**3**
		Statistics with study removed	1.58	0.10	<0.0001	-91.74%	
	5/6SNX+Dox-HF	**Overall effect**	**1.04**	**0.52**	**=0.045**	**-**	**3**
		Statistics with study removed	1.54	0.49	=0.0015	-6.52%	-
			1.05	1.00	NS	93.55%	
	ACF	**Overall effect**	**3.26**	**0.76**	**<0.0001**	**-**	**3**
		Statistics with study removed	4.12	0.97	<0.0001	28.51%	
	Dahl/SS	**Overall effect**	**0.83**	**0.60**	**NS**	**-**	**5**
		Statistics with study removed	0.34	0.33	NS	-44.66%	
			0.90	0.87	NS	44.85%	
	MI (LAD-MI)	**Overall effect**	**2.75**	**0.24**	**<0.0001**	**-**	**17**
		Statistics with study removed	2.81	0.27	<0.0001	13.09%	
	Ren-2Tg	**Overall effect**	**0.22**	**0.39**	**NS**	**-**	**3**
		**Statistics with study removed**	**0.08**	**0.49**	**NS**	**25.95%**	
			**0.57**	**0.11**	**<0.0001**	**-70.56%**	
	Ren-2Tg+ACF	**Overall effect**	**3.54**	**0.51**	**<0.0001**	**-**	**3**
		Statistics with study removed	3.39	0.71	<0.0001	40.16%	
			4.05	0.11	<0.0001	-79.29%	
	TAC	**Overall effect**	**0.59**	**0.13**	**<0.0001**	**-**	**8**
		Statistics with study removed	0.17	0.04	<0.0001	-66.85%	
			0.64	0.28	=0.02	115.37%	
**LVESP [mmHg] (D, difference in means)**	5/6SNX	**Overall effect**	**17.93**	**6.35**	**=0.005**	**-**	**6**
		Statistics with study removed	16.71	7.70	=0.03	21.19%	
			13.24	4.76	=0.005	-25.02%	
	ISO-HF	**Overall effect**	**-31.09**	**1.50**	**<0.0001**	**-**	**4**
		Statistics with study removed	-30.20	2.06	<0.0001	37.07%	
	MI (LAD-MI)	**Overall effect**	**-13.90**	**2.22**	**<0.0001**	**-**	**9**
		Statistics with study removed	-14.19	2.70	<0.0001	21.40%	
			-12.20	1.95	<0.0001	-12.23%	
**Myocardial hypertrophy (R, response ratio)**	2K1C	**Overall effect**	**1.33**	**0.09**	**=0.0001**	**-**	**6**
		Statistics with study removed	1.31	0.11	=0.002	20.27%	
			1.40	0.05	<0.0001	-50.27%	
	5/6SNX	**Overall effect**	**1.23**	**0.03**	**<0.0001**	**-**	**41**
		Statistics with study removed	1.23	0.03	<0.0001	4.02%	
			1.21	0.03	<0.0001	-5.56%	
	5/6SNX+MI (LAD-MI)	**Overall effect**	**1.16**	**0.04**	**<0.0001**	**-**	**9**
		Statistics with study removed	1.18	0.05	=0.0001	22.08%	
			1.12	0.04	=0.001	-4.78%	
	ACF	**Overall effect**	**1.62**	**0.15**	**<0.0001**	**-**	**14**
		Statistics with study removed	0.21	1.29	=0.0001	36.84%	
	ARF	**Overall effect**	**1.35**	**0.05**	**<0.0001**	**-**	**5**
		Statistics with study removed	1.34	0.09	<0.0001	68.61%	
			1.41	0.03	<0.0001	-36.94%	
	bilateral R-IR	**Overall effect**	**1.16**	**0.05**	**=0.0006**	**-**	**8**
		Statistics with study removed	1.17	0.06	=0.001	12.38%	
			1.14	0.05	=0.003	-3.04%	
	Dahl/SS	**Overall effect**	**1.49**	**0.11**	**<0.0001**	**-**	**17**
		Statistics with study removed	1.52	0.12	<0.0001	10.65%	
			1.54	0.10	<0.0001	-11.05%	
	ISO-HF	**Overall effect**	**1.36**	**0.04**	**<0.0001**	**-**	**8**
		Statistics with study removed	1.37	0.05	<0.0001	12.81%	
			1.39	0.03	<0.0001	-34.52%	
		**Overall effect**	**1.25**	**0.03**	**<0.0001**	**-**	**37**

	MI (LAD-MI)	Statistics with study	1.25	0.03	<0.0001	8.44%	
		removed	1.25	0.02	<0.0001	-18.40%	
	Ren-2Tg	**Overall effect**	**1.21**	**0.05**	**<0.0001**	**-**	**10**
		Statistics with study	1.20	0.08	<0.0001	57.06%	
		removed	1.26	0.03	<0.0001	-35.85%	
	Ren-2Tg+ACF	**Overall effect**	**1.65**	**0.05**	**<0.0001**	**-**	**8**
		Statistics with study	1.68	0.07	<0.0001	43.65%	
		removed	1.61	0.05	<0.0001	-5.82%	
	SHR	**Overall effect**	**1.20**	**0.08**	**=0.009**	**-**	**4**
		Statistics with study	1.19	0.10	<0.0001	24.35%	
		removed	1.25	0.05	<0.0001	-44.44%	
	SHR-stroke prone	**Overall effect**	**1.56**	**0.12**	**<0.0001**	**-**	**4**
		Statistics with study	1.71	0.16	<0.0001	34.62%	
		removed	1.44	0.12	<0.0001	-0.96%	
	STZ	**Overall effect**	**1.56**	**0.19**	**=0.0003**	**-**	**8**
		Statistics with study	1.63	0.23	=0.0007	24.21%	
		removed	1.33	0.11	<0.0001	-40.23%	
	TAC	**Overall effect**	**1.52**	**0.07**	**<0.0001**	**-**	**11**
		Statistics with study	1.52	0.08	<0.0001	21.66%	
		removed	1.48	0.06	<0.0001	-8.29%	
	unilateral R-IR	**Overall effect**	**1.18**	**0.03**	**<0.0001**	**-**	**4**
		Statistics with study	1.19	0.05	<0.0001	69.23%	
		removed	1.18	0.02	<0.0001	-48.44%	
	uninephrectomized DOCA	**Overall effect**	**1.33**	**0.02**	**<0.0001**	**-**	**16**
		Statistics with study	1.33	0.02	<0.0001	51.34%	
		removed	1.34	0.01	<0.0001	-21.63%	
	UNX+ACF	**Overall effect**	**1.53**	**0.09**	**<0.0001**	**-**	**4**
		Statistics with study removed	1.58	0.12	<0.0001	29.08%	
**SBP [mmHg] (D, difference in means)**	2K1C	**Overall effect**	**77.47**	**8.55**	**<0.0001**	**-**	**6**
		Statistics with study	77.18	10.81	<0.0001	26.46%	
		removed	71.00	7.79	<0.0001	-8.91%	
	5/6SNX	**Overall effect**	**39.93**	**5.20**	**<0.0001**	**-**	**37**
		Statistics with study	40.54	5.35	<0.0001	2.82%	
		removed	38.68	4.62	<0.0001	-11.05%	
	5/6SNX+MI (LAD-MI)	**Overall effect**	**10.04**	**8.14**	**NS**	**-**	**6**
		Statistics with study	12.47	11.70	NS	43.72%	
		removed	1.81	7.11	NS	-12.70%	
	ARF	**Overall effect**	**14.58**	**14.37**	**NS**	**-**	**3**
		Statistics with study	11.55	18.50	NS	28.73%	
		removed	6.34	13.98	NS	-2.69%	
	Dahl/SS	**Overall effect**	**62.76**	**9.25**	**<0.0001**	**-**	**17**
		Statistics with study	61.58	10.63	<0.0001	14.93%	
		removed	61.98	9.86	<0.0001	-6.56%	
	MI (LAD-MI)	**Overall effect**	**-6.22**	**1.70**	**=0.0003**	**-**	**18**
		Statistics with study	-6.56	1.81	<0.0001	6.17%	
		removed	-6.77	1.57	<0.0001	-7.44%	
	SHR	**Overall effect**	**68.66**	**18.13**	**=0.0002**	**-**	**4**
		Statistics with study	60.07	22.08	=0.006	21.81%	
		removed	58.75	13.67	<0.0001	-24.61%	
	SHR-stroke prone	**Overall effect**	**110.84**	**11.68**	**<0.0001**	**-**	**3**
		Statistics with study	106.04	22.00	<0.0001	88.33%	
		removed	123.20	3.92	<0.0001	-66.46%	
	STZ	**Overall effect**	**17.14**	**11.46**	**NS**	**-**	**6**
		Statistics with study removed	13.84	14.95	NS	30.47%	
	TAC	**Overall effect**	**-0.66**	**1.63**	**NS**	**-**	**5**
		Statistics with study	-0.91	2.17	NS	33.41%	
		removed	1.15	1.36	NS	-16.30%	
	uninephrectomized DOCA	**Overall effect**	**45.36**	**4.15**	**<0.0001**	**-**	**13**
		Statistics with study	45.83	4.98	<0.0001	20.02%	
		removed	43.82	4.00	<0.0001	-3.65%	
**S-Cre [mg/dl]**	2K1C	**Overall effect**	**0.26**	**0.07**	**=0.0001**	**-**	**7**
		Statistics with study	0.24	0.09	=0.009	34.77%	
		removed	0.32	0.06	<0.0001	-11.71%	

**(D, difference in means)**	5/6SNX	**Overall effect**	**0.56**	**0.04**	**<0.0001**	**-**	**55**
		Statistics with study removed	0.57	0.04	<0.0001	6.32%	
			0.55	0.03	<0.0001	-3.35%	
	5/6SNX+MI (LAD-MI)	**Overall effect**	**0.78**	**0.13**	**<0.0001**	**-**	**9**
		Statistics with study removed	0.88	0.15	<0.0001	14.38%	
			0.77	0.11	<0.0001	-15.19%	
	5/6SNX+Dox-HF	**Overall effect**	**1.03**	**0.17**	**<0.0001**	**-**	**6**
		Statistics with study removed	1.18	0.31	<0.0001	83.95%	
			0.84	0.10	<0.0001	-39.89%	
	ACF	**Overall effect**	**0.01**	**0.01**	**NS**	**-**	**3**
		Statistics with study removed	0.01	0.01	NS	37.34%	
	ARF	**Overall effect**	**2.42**	**0.96**	**=0.012**	**-**	**5**
		Statistics with study removed	2.64	1.10	=0.016	14.75%	
			1.90	0.43	<0.0001	-55.60%	
	bilateral R-IR	**Overall effect**	**1.33**	**0.18**	**<0.0001**	**-**	**11**
		Statistics with study removed	1.31	0.29	<0.0001	61.06%	
			1.24	0.15	<0.0001	-13.31%	
	Dahl/SS	**Overall effect**	**0.09**	**0.02**	**<0.0001**	**-**	**11**
		Statistics with study removed	0.09	0.03	<0.0001	28.00%	
			0.08	0.02	=0.0002	-9.50%	
	Dox-HF	**Overall effect**	**0.57**	**0.18**	**=0.0013**		**4**
		Statistics with study removed	0.59	0.26	<0.0001	44.40%	
			0.66	0.06	=0.0002	-66.50%	
	ISO-HF	**Overall effect**	**0.18**	**0.18**	**NS**	**-**	**5**
		Statistics with study removed	0.22	0.22	NS	19.81%	
			0.02	0.01	NS	-94.61%	
	MI (LAD-MI)	**Overall effect**	**0.13**	**0.01**	**<0.0001**	**-**	**28**
		Statistics with study removed	0.13	0.02	<0.0001	7.81%	
			0.10	0.01	<0.0001	-7.71%	
	M-IR	**Overall effect**	**0.58**	**0.27**	**=0.03**	**-**	**4**
		Statistics with study removed	0.74	0.33	=0.024	20.98%	
	STZ	**Overall effect**	**0.43**	**0.12**	**=0.0005**	**-**	**6**
		Statistics with study removed	0.50	0.15	=0.0008	19.97%	
			0.41	0.12	=0.0008	-1.35%	
	TAC	**Overall effect**	**0.01**	**0.07**	**NS**	**-**	**5**
		**Statistics with study removed**	**0.01**	**0.09**	**NS**	**21.88%**	
			**-0.06**	**0.03**	**=0.01**	**64.95%**	
	unilateral R-IR	**Overall effect**	**0.70**	**0.47**	**NS**	**-**	**4**
		Statistics with study removed	0.91	0.81	NS	74.40%	
			0.15	0.25	NS	-45.63%	
	uninephrectomized DOCA	**Overall effect**	**0.23**	**0.07**	**=0.0008**	**-**	**3**
		Statistics with study removed	0.25	0.09	=0.005	27.94%	
			0.16	0.01	=0.0008	-80.34%	
**Renal fibrosis (R, response ratio)**	5/6SNX	**Overall effect**	**4.26**	**0.79**	**<0.0001**	**-**	**11**
		Statistics with study removed	4.70	1.16	<0.0001	46.41%	
			3.95	0.68	<0.0001	-14.18%	
	5/6SNX+(LAD)MI	**Overall effect**	**2.98**	**1.09**	**=0.003**	**-**	**3**
		Statistics with study removed	4.34	1.73	=0.0002	58.88%	
	Dahl/SS	**Overall effect**	**2.28**	**0.38**	**<0.0001**	**-**	**5**
		Statistics with study removed	2.23	0.55	=0.001	42.20%	
			2.64	0.18	<0.0001	-53.96%	
	ISO-HF	**Overall effect**	**6.17**	**1.91**	**<0.0001**	**-**	**4**
		Statistics with study removed	7.16	2.87	=0.001	50.50%	
			4.39	0.99	<0.0001	-47.86%	
	MI (LAD-MI)	**Overall effect**	**2.19**	**0.45**	**=0.0001**	**-**	**8**
		Statistics with study removed	2.44	0.69	=0.0014	52.45%	
			1.64	0.20	=0.0001	-54.61%	
	STZ	**Overall effect**	**2.42**	**0.43**	**=0.0001**	**-**	**3**
		Statistics with study removed	1.99	0.20	=0.001	-53.81%	
		**Overall effect**	**13.88**	**11.90**	**=0.002**	**-**	**3**
			11.36	12.22	=0.02	2.74%	

	uninephrectomized DOCA	Statistics with study removed	8.46	7.22	=0.01	-39.35%	
**UAlbEx [mg/24h] (D, difference in means)**	5/6SNX	**Overall effect**	**65.71**	**1.78**	**<0.0001**	**-**	**11**
		Statistics with study removed	77.87	1.95	<0.0001	10.05%	
			12.67	0.91	<0.0001	-48.61%	
	ACF	**Overall effect**	**5.14**	**2.50**	**=0.04**	**-**	**3**
		Statistics with study removed	2.90	0.02	<0.0001	-99.00%	
	Dahl/SS	**Overall effect**	**88.45**	**42.64**	**=0.04**	**-**	**3**
		Statistics with study removed	110.71	20.99	<0.0001	-50.78%	
	MI (LAD-MI)	**Overall effect**	**0.35**	**0.12**	**=0.004**	**-**	**5**
		Statistics with study removed	0.32	0.14	=0.02	10.24%	
**UProtEx [mg/24h] (D, difference in means)**	5/6SNX	**Overall effect**	**136.09**	**11.26**	**<0.0001**	**-**	**18**
		Statistics with study removed	146.38	12.79	<0.0001	13.52%	
			143.49	10.65	<0.0001	-5.54%	
	5/6SNX+MI (LAD-MI)	**Overall effect**	**46.05**	**3.76**	**<0.0001**	**-**	**7**
		Statistics with study removed	61.31	4.29	<0.0001	14.12%	
			9.22	2.11	<0.0001	-43.78%	
	Dahl/SS	**Overall effect**	**512.59**	**145.12**	**<0.0001**	**-**	**3**
		Statistics with study removed	479.80	210.50	=0.02	45.05%	
			636.86	55.74	<0.0001	-61.59%	
	MI (LAD-MI)	**Overall effect**	**12.46**	**4.42**	**=0.005**	**-**	**13**
		Statistics with study removed	13.44	6.19	=0.03	39.97%	
	SHR-stroke prone	**Overall effect**	**61.40**	**26.04**	**=0.018**	**-**	**5**
		Statistics with study removed	63.84	30.83	=0.038	18.40%	
			49.62	21.02	=0.018	-19.26%	
	UNX+MI	**Overall effect**	**83.13**	**58.38**	**NS**	**-**	**3**
		Statistics with study removed	71.05	73.85	NS	26.50%	
			51.55	55.24	NS	-5.38%	
**BW[g] (D, difference in means)**	2K1C	**Overall effect**	**-65.84**	**35.46**	**NS**		**3**
		**Statistics with study removed**	**-58.51**	**64.1**	**NS**	**80.75%**	
			**-101.47**	**21.20**	**<0.0001**	**-40.22%**	
	5/6SNX	**Overall effect**	**-30.54**	**4.17**	**<0.0001**	-	**30**
		Statistics with study removed	-31.39	4.72	<0.0001	13.42%	
			-28.87	3.63	<0.0001	-12.8%	
	5/6SNX+MI (LAD-MI)	**Overall effect**	**-20.98**	**10.22**	**=0.04**	**-**	**4**
		**Statistics with study removed**	**-33.93**	**18.55**	**NS**	**81.58%**	
			**-5.48**	**6.51**	**NS**	**-36.29%**	
	ACF	**Overall effect**	**6.87**	**10.53**	**NS**	**-**	**11**
		Statistics with study removed	10.22	14.49	NS	37.54%	
			16.42	8.67	NS	-17.73%	
	ARF	**Overall effect**	**-43.35**	**5.98**	**<0.0001**		**5**
		Statistics with study removed	-20.81	4.83	<0.0001	-19.25%	
			-49.39	6.66	<0.0001	10.67%	
	Dahl/SS	**Overall effect**	**-55.56**	**9.59**	**<0.0001**	**-**	**19**
		Statistics with study removed	-55.65	11.67	<0.0001	21.69%	
			-50.80	8.195	<0.0001	40.51%	
	ISO-HF	**Overall effect**	**-37.75**	**6.57**	**<0.0001**	**-**	**4**
		Statistics with study removed	-36.97	9.56	=0.0001	45.44%	
			-42.48	3.00	<0.0001	-54.30%	
	MI (LAD-MI)	**Overall effect**	**-3.91**	**1.31**	**=0.003**	-	**33**
		Statistics with study removed	-3.42	1.40	=0.009	-0.12%	
			-4.40	1.51	=0.003	15.44%	
	Ren-2Tg	**Overall effect**	**46.33**	**27.58**	**NS**	-	**7**
		Statistics with study removed	57.39	37.61	NS	36.35%	
			8.30	8.27	NS	-70.02%	
	Ren-2Tg+ACF	**Overall effect**	**-29.18**	**11.22**	**=0.009**	**-**	**4**
		Statistics with study removed	-37.06	17.13	=0.03	52.65%	
			-13.65	4.70	=0.004	-58.11%	
	SHR	**Overall effect**	**-10.86**	**16.83**	**NS**		**3**
			-14.40	29.49	NS	75.21%	

		Statistics with study removed	5.69	9.49	NS	-43.59%	
	SHR-stroke prone	**Overall effect**	**-57.05**	**42.03**	**NS**	**-**	**4**
		**Statistics with study removed**	**-77.58**	**66.60**	**NS**	**58.45%**	
			**-13.30**	**2.71**	**<0.0001**	**-93.55%**	
	STZ	**Overall effect**	**-87.25**	**12.71**	**<0.0001**		**10**
		Statistics with study removed	-93.65	23.24	<0.0001	82.83%	
	TAC	**Overall effect**	**0.06**	**0.68**	**NS**	-	**8**
		Statistics with study removed	0.14	0.92	NS	34.09%	
			-0.05	0.37	NS	-46.64%	
	unilateral R-IR	**Overall effect**	**-1.21**	**0.44**	**=0.006**	-	**3**
		Statistics with study removed	-1.56	0.56	=0.005	26.17%	
			-0.76	0.34	=0.02	23.30%	
	uninephrectomized DOCA	**Overall effect**	**-20.14**	**2.06**	**<0.0001**	**-**	**10**
		Statistics with study removed	-23.84	2.96	<0.0001	43.67%	
			-11.87	1.49	<0.0001	-27.68%	

* – statistics after excluding the study displaying the most impact (change in standard error (+/-)) on the overal result; ** – at least three interventions must have been included into analysis, in relation to individual parameter and animal model. Mostly, the selected studies included into analysis did not affect the pooled results with regard to individual parameter. The exception concerned the alternations in EF parameter that were observed in ARF model, LVESd – in 5/6SNX or Ren-2Tg animals, S-Cre – in TAC subjects, LVEDP – in uninephrectomized animals exposed to coronary artery ligation, body weight for 2K1C subjects and 5/6SNX+MI (LAD-MI) model. 2K1C – 2-kidney 1-clip; ACF – aorto-caval fistula; ACR – albumin to creatinine urinary excretion ratio; ARF – adenine-induced renal failure; BUN – blood urea nitrogen; BW – body weight; DOCA-salt – deoxycorticosterone acetate; Dox-HF – doxorubicin-induced heart failure; dP/dt_{max} – maximal rate of pressure increase; dP/dt_{min} – maximal rate of pressure decrease; EF – left ventricle ejection fraction; FS – fractional shortening; GFR – glomerular filtration rate; GK – Goto-Kakizaki; ISO-HF – isoproterenol-induced HF; LAD-MI – left anterior descending coronary artery ligation; LVEDd – left ventricular end-diastolic diameter; LVEDP – left ventricular end-diastolic pressure; LVESd – left ventricular end-systolic diameter; LVESP – left ventricular end-systolic pressure; Ren-2 Tg – (mRen2)27 transgenic; R-IR – renal ischemia-reperfusion; SBP – systolic blood pressure; S-Cre – serum creatinine; SHR – spontaneous hypertensive rat; SNX – subtotal nephrectomy; STZ – streptozotocin injected; TAC – transverse aortic constriction; UAlbEx – urinary albumin excretion; UNX – unilateral nephrectomy; UProtEx – urinary protein excretion; UUO – unilateral urinary obstruction.

Supplementary. S3 Table. Study characteristics.

1st author	Animal model	Species	Race (genetic back-ground)	Sex	Age (wks)	Intervention	Comparison	Ref
Adel M	3/4 SNX+ISO	rat	SD			100 mg/kg, twice (ISO)	Sham operated+placebo	[6]
Akinrinde AS	CoCl2-HF	rat	Wistar	male	8-10	350 ppm for 7 days in drinking water	Placebo	[7]
Altara R	Dahl/SS	rat		male	6	HS, 4% NaCl	LS, 0.3% NaCl	[8]
Amador-Martínez I	5/6 SNX	mouse	C57BL/6	male	8		Sham operated	[9]
Amador-Martínez I	bilateral R-IR	rat	Wistar	male	11	45 min.	Sham operated	[10]
Arozal W	STZ	rat	SD	male	8	55 mg/kg, ip	Placebo	[11]
Baccam GC	5/6 SNX	mouse	CD1	male	10		Sham operated	[12]
Barone FC	SHR stroke-prone	rat		male	11.5		WKY	[13]
Beikoghli Kalkhoran S	ARF	rat	Wistar	male	9	0.3% chow, 8 weeks	Placebo	[14]

Beraldo JI	5/6 SNX	rat	Wistar	male	8		Sham operated	[15]
Biala A	Dahl/SS	rat		male	7	HS, 8% NaCl	LS, 0.3% NaCl	[16]
Bigelman E	5/6 SNX	rat	Lewis	male			Sham operated	[17]
Bishara B	ACF	rat	SD	male			Sham operated	[18]
Bryan PM	TAC	mouse	C57	male			Sham operated	[19]
Burrell LM	LAD-MI	rat	SD	female			Sham operated	[20]
Cai C	bilateral R-IR	mouse	FUNDC1 knockout			30 min., 72h reperfusion	Sham operated	[21]
Campbell DJ	STZ	rat	SD	female	5	55 mg/kg, ip	Placebo	[22]
Carvalho LRRA	UNX+WD	mouse	C57BL/6J	male			Sham operated	[23]
Casellas D	SHR+L-NAME	rat	SHR	male		20 mg/kg, per os, 10 days	Placebo	[24]
Červenka L	ACF	rat	HanSD	male	9		Sham operated	[25]
Chaihongsa N	2K1C	rat	SD	male			Sham operated	[26]
Chang D	5/6 SNX	rat	SD	male			Sham operated	[27]
Chang D	5/6 SNX	rat	SD	male			Sham operated	[28]
Chang Y	UUO	rat	Wistar	male			Sham operated	[29]
Chen G	UUO	rat	Wistar	male			Sham operated	[30]
Chen K	PAC	mouse	C57BL/6	male	7		Sham operated	[31]
Chen KH	5/6 SNX	rat	SD	male			Sham operated	[32]
Chen P	TAC	rat	SD		10		Sham operated	[33]
Chen TH	bilateral R-IR	rat	SD			45min, 3 h reperfusion	Sham operated	[34]
Chen X	ISO-HF	rat	SD	male	6	5 mg/kg, sc one week	Placebo	[35]
Chen X	5/6 SNX	rat	Wistar	male	7		Sham operated	[36]
Cheng ZJ	GK	rat		male	8		Wistar	[37]
Cho E	LAD-MI	rat	SD	male			Sham operated	[38]
Chu PY	uninephrectomized DOCA	mouse	C57BL/6	male	6	SR pellet – sc containing 150 mg, 8 wks	Sham operated+placebo	[39]
Chua S	5/6 SNX+Dox-HF	rat	SD	male		7 mg/kg every five days/20 days (Dox)	Sham operated+placebo	[40]
Chuppa S	5/6 SNX	rat	SD	male	8		Sham operated	[41]
Cohen-Segev R	ACF	rat	SD	male			Sham operated	[42]
de Resende MM	LAD-MI	rat	Wistar	male			Sham operated	[43]
Dikow R	5/6 SNX	rat	SD	male	20		Sham operated	[44]
Ding SS	MI	rat	Wistar	male			Sham operated	[45]

Dionísio LM	5/6 SNX	rat	Wistar	male			Sham operated	[46]
Dizaye K	ISO-HF	rat	Wistar	female	20	5 mg/kg, ip for 1 week	Placebo	[47]
Dominguez JH	bilateral R-IR	rat	SD	male		30 min.	Sham operated	[48]
Dong X	5/6 SNX	mouse	C57BL/6	male	8		Sham operated	[49]
Dong Z	LAD-MI	rat	SD	male	24		Sham operated	[50]
Duchesne R	5/6 SNX	rat	SD				Sham operated	[51]
Elmahallawy EK	STZ	rat	Wistar	male	6	45 mg/kg, ip	Placebo	[52]
Enes ALT	2K1C	rat	Wistar	male			Sham operated	[53]
Entin-Meer M	5/6 SNX	rat	Lewis	male			Sham operated	[54]
Falconi CA	unilateral R-IR	mouse	C57BL/6	male	7	60 min.	Sham operated	[55]
Feng Q	MI	rat	SD	male			Sham operated	[56]
Fontes MS	DOCA-salt	mouse	129/Sv	male	30	pellets with 3.3 mg/day (DOCA)+3% NaCl, 8 wks, 26wks 6%NaCl	Placebo	[57]
Francis J	MI	rat	Harlan SD	male			Sham operated	[58]
Fukuoka K	5/6 SNX	rat	ICR	male	7		Sham operated	[59]
Furcea DM	Dox-HF	rat	Wistar	male	7	1 mg/kg, ip twice a week for 6 wks	Placebo	[60]
Gallego-Delgado J	UNX+SHR	rat		male	12		Sham operated WKY	[61]
Gawrys O	Ren-2 Tg	rat	HanSD	male	9		HanSD	[62]
Goldfarb M	ACF	rat	SD				Sham operated	[63]
Goltsman I	ACF	rat	SD	male	10		Sham operated	[64]
Goltsman I	ACF	rat	SD	male			Sham operated	[65]
Gonzalez L	uninephrectomized DOCA	rat	SD	male		60 mg, im two times per week, 4 wks	Sham operated+placebo	[66]
Goyal BR	STZ	rat	Wistar	female		45 mg, ip	Placebo	[67]
Guo H	Dahl/SS+SOD 3E124D	rat			8	HS, 8% NaCl	LS, 0.3% NaCl	[68]
Guo J	5/6 SNX	mouse	C57BL/6	male			Sham operated	[69]
Habeichi NJ	LAD-MI	mouse	C57BL6/J	both			Sham operated	[70]
Halade GV	LAD-MI	mouse	C57BL/6	male			Sham operated	[71]
Ham O	UUO	mouse	C57BL/6	male	10		Sham operated	[72]
Hamzaoui M	5/6 SNX	mouse	129/Sv	male			Sham operated	[73]
Han X	bilateral R-IR	mouse	C57BL/6J	male	9	40 min.	Sham operated	[74]

Hao H	LAD-MI	mouse	C57BL/6	male	10		Sham operated	[75]
Hao PP	STZ	rat	Wistar	male	5	No data	Placebo	[76]
Harrison JC	LAD-MI	rat	Lewis	male			Sham operated	[77]
Hirsch AT	LAD-MI	rat	SD	male	8		Sham operated	[78]
Homma T	UNX	rat	Wistar	male	6		Sham operated	[79]
Honetschlagerová Z	ACF	rat	FHH	male	12		Sham operated FHL	[80]
Honetschlägerová Z	Ren-2 Tg	rat	HanSD	male			HanSD	[81]
Hong MH	2K1C	rat	HanSD	male	8		Sham operated	[82]
Hong MH	ISO-HF	rat	SD	male	6	10 mg, ip 4 wks	Placebo	[83]
Huang D	5/6 SNX	mouse	C57BL/6	male	8		Sham operated	[84]
Iampanichakul M	2K1C	rat	SD	male	5		Sham operated	[85]
Ikeda T	Dahl/SS	rat		male	6	HS, 8% NaCl	LS, 0.3% NaCl	[86]
Ikenaga H	MI	rat	SD	male			Sham operated	[87]
Inada Y	SHR stroke-prone+4%NaCl	rat	WKY	male	6		WKY	[88]
Ishikawa M	Dahl/SS	rat		male	6	HS, 8% NaCl	Dahl/SR	[89]
Ishimitsu T	SHR	rat	SHR	male	15		WKY	[90]
Ito D	LAD-MI	rat	SD	male	8		Sham operated	[91]
Ito H	Dahl/SS	rat		male	8	HS, 4% NaCl	LS, 0.3% NaCl	[92]
Jíchová Š	Ren-2 Tg	rat	HanSD	male	9		HanSD	[93]
Jin L	LAD-MI	mouse	C57BL/6	male	8		Sham operated	[94]
Junho CVC	unilateral R-IR	mouse	C57BL/6	male	8	60 min.	Sham operated	[95]
Junho CVC	unilateral R-IR	mouse	C57BL/6	male	8	60 min.	Sham operated	[96]
Kain V	MI	mouse	C57BL/6	male	7		Sham operated	[97]
Kain V	unilateral R-IR	mouse	C57BL/6		10		Sham operated	[98]
Kala P	5/6 SNX+ACF	rat	HanSD	male	8		Sham operated	[99]
Kala P	Ren-2 Tg	rat	HanSD	male	9		HanSD	[100]
Kala P	Ren-2 Tg	rat	HanSD	male	9		HanSD	[101]
Kashioulis P	ARF	rat	SD	male		Chow conaining 0.15% – 0.5% adenine, 9 wks	Placebo	[102]
Katsurada K	MI	rat	SD	male			Sham operated	[103]
Katsurada K	MI	rat	SD	male			Sham operated	[104]
Keihanian F	ISO-HF	rat	Wistar	male	16	10 mg/kg, sc 10 days	Placebo	[105]

Kieswich JE	ARF	mouse	C57BL/6	male	8	Chow containing 0.15% adenine, 20 wks	Placebo	[106]
Koc K	unilateral R-IR	rat	Wistar	female		50 min., 3h	Sham operated	[107]
Koch V	5/6 SNX	rat	Wistar	male	4		Sham operated	[108]
Kolkhof P	uninephrectomized DOCA	rat	HanSD	male		30 mg, sc once a week, 10 wks	Sham operated+placebo	[109]
Kratky V	ACF	rat	HanSD	male			Sham operated	[110]
Kratky V	Ren-2 Tg	rat	HanSD	male			HanSD	[111]
Krishnan V	MI	mouse		male			Sham operated	[112]
Kuczmarski JM	5/6 SNX	rat	SD	male	12		Sham operated	[113]
Langlo KAR	Dahl/SS	rat		male	9	HS, 8% NaCl	LS, 0.3% NaCl	[114]
Lekawanvijit S	LAD-MI	rat	SD	male			Sham operated	[115]
Lekawanvijit S	LAD-MI	rat	SD	male			Sham operated	[116]
Li JD	ISO-HF	mouse	C57BL/6	male	10	40 mg/kg, sc 2 wks	Placebo	[117]
Li Y	5/6 SNX	mouse	C57BL/6J	male	12		Sham operated	[118]
Li Y	uninephrectomized DOCA	mouse	C57BL/6J	male	12	2.4 mg/day, sc 3 wks	Sham operated+placebo	[119]
Li Y	5/6 SNX	rat	Wistar	male			Sham operated	[120]
Lima Posada I	5/6 SNX	rat	SD	male	6		Sham operated	[121]
Lin HC	STZ	rat	SD	male	5	55 mg/kg, ip	Placebo	[122]
Lindoso RS	uninephrectomized DOCA	rat	Wistar	male	7	8 mg/kg, sc twice a week	Sham operated+placebo	[123]
Liu B	5/6 SNX	mouse	C57BL/6J	male	5		Sham operated	[124]
Liu Q	ISO-HF	rat	Wistar	male		5 mg/kg, ip 5 wks	Placebo	[125]
Liu S	5/6 SNX	rat	SD	male			Sham operated	[126]
Liu S	5/6 SNX, 5/6 SNX+ LAD-MI	rat	SD	male			Sham operated	[127]
Liu T	5/6 SNX	mouse	C57BL/6J	male	8		Sham operated	[128]
Liu Y	5/6 SNX	rat	SD	male			Sham operated	[129]
Lother A	uninephrectomized DOCA	mouse			12	2.5 mg, sc 6 wks	Sham operated+placebo	[130]
Luettges K	uninephrectomized DOCA	mouse	C57BL6/J	male		2.4 mg/day, sc	Sham operated+placebo	[131]
Lun MH	M-IR	rat	SD			60 min, 2h	Sham operated	[132]
Marques FZ	uninephrectomized DOCA	mouse	C57Bl/6	male	6	3 weeks	Sham operated+placebo	[133]

Matsuura R	TAC	mouse	C57BL/6	male	8		Sham operated	[134]
Meagher P	GK	rat	Wistar	male	12		Wistar	[135]
Melenovsky V	ACF	rat	Wistar	male			Sham operated	[136]
Miloradović Z	unilateral R-IR	rat	Wistar	male			Sham operated	[137]
Mittal A	RAL	rat	Wistar	male	20		Sham operated	[138]
Moench I	5/6 SNX	rat	Wistar	male			Sham operated	[139]
Mohamed RMSM	5/6 SNX	rat	Wistar	male			Sham operated	[140]
Monu SR	LAD-MI	mouse	C57	male	9		Sham operated	[141]
Moubarak M	2K1C	rat	Wistar	male			Sham operated	[142]
Muromachi N	UNX+AngII	mouse	C57BL/6J	male	8	1.2 mg, one week	Sham operated+placebo	[143]
Nagasu H	UNX	mouse	C57BL/6J	male	8		Sham operated	[144]
Nakajima Y	Dahl/SS	rat		male	6.5	HS, 8% NaCl	LS, 0.3% NaCl	[145]
Nakano S	ARF	rat	Wistar	male	7	600 mg, 10 days	Placebo	[146]
Nakatsukasa T	Dahl-Iwai S	rat		male	6	HS, 8% NaCl	LS, 0.3% NaCl	[147]
Namba M	Dahl/SS	rat		male	6	HS, 8% NaCl	LS, 0.3% NaCl	[148]
Nanto-Hara F	ARF	mouse	C57BL/6	male	8	0.2%, per os 6 wks	Placebo	[149]
Nasci VL	5/6 SNX	rat	SD	male			Sham operated	[150]
Ni SH	5/6 SNX	mouse	C57BL/6	male	6		Sham operated	[151]
Nishikimi T	Dahl/SS	rat		male	6	HS, 8% NaCl	Dahl/SR	[152]
Nishikimi T	SHR	rat		male	9		WKY	[153]
Noiri E	Dox-HF	rat	SD	male	7	3 mg, 4 wks	Placebo	[154]
Novoa U	STZ	mouse	Balb/c			100, 100, and 200 mg/kg, ip	Placebo	[155]
Nozawa T	MI	rat	Wistar	male			Sham operated	[156]
Ogawa M	LAD-MI	mouse	C57BL/6	male	6		Sham operated	[157]
Ogawa T	DOCA-salt+1%NaCl	rat	SD	male		30 mg/kg, sc once a week, 5 wks	Placebo	[158]
Ohno K	LAD-MI	rat		male	25		Sham operated	[159]
Ola-Davies OE	Bisphenol A	rat		male		20 mg/kg, 2 wks	Placebo	[160]
Omizo H	5/6 SNX+oxonic acid (OA)	rat	SD	male	6	2g/100g chow (OA)	Sham operated+placebo	[161]
Otero-Losada M	HSD	rat	Wistar	male		commercially available sucrose-sweetened carbonated	Placebo	[162]

						drink, Coca-Cola		
Oudot C	Dahl/SS	rat		male	6	HS, 8% NaCl	LS, 0.26% NaCl	[163]
Ozoux ML	Dahl/SS	rat		male		HS, 2% NaCl	LS, 0.3% NaCl	[164]
Palygin O	GK	rat		male	48		Wistar	[165]
Pan Y	LAD-MI	mouse	C57BL/6	male	8		Sham operated	[166]
Paulin FV	2K1C	rat	Wistar	male	13		Sham operated	[167]
Pesce P	LAD-MI	mouse	CD1				Sham operated	[168]
Phyu HE	STZ	rat	Wistar	male	10	65 mg/kg, ip	Placebo	[169]
Poveda J	bilateral R-IR	mouse			13	45 min.	Sham operated	[170]
Prado NJ	UUO	rat	WKY	male			Sham operated	[171]
Prem PN	bilateral R-IR	rat	Wistar	male		45 min.	Sham operated	[172]
Qiu C	MI	rat	Wistar	male			Sham operated	[173]
Ribeiro S	5/6 SNX	rat	Wistar	male	12		Sham operated	[174]
Rofe MT	5/6 SNX+LAD-MI	rat	Lewis				Sham operated	[175]
Sainsily X	SHR	rat	SHR	male	10		WKY	[176]
Saito T	uninephrectomized DOCA	mouse	C57BL/6	male	8	50 mg, sc 7 days	Placebo	[177]
Saliba Y	2K1C	rat	Wistar	male			Sham operated	[178]
Sárközy M	5/6 SNX	rat	Wistar	male	9		Sham operated	[179]
Sárközy M	5/6 SNX	rat	Wistar	male			Sham operated	[180]
Savira F	LAD-MI	rat	SD	male	8		Sham operated	[181]
Sayour AA	M-IR	rat	SD	male	7	30min, 2h reperfusion	Sham operated	[182]
Sharkovska Y	5/6 SNX	rat	Wistar	male	9		Sham operated	[183]
Sharma AK	STZ	rat		both		55 mg/kg, ip	Placebo	[184]
Sharma G	STZ+HFD	rat	Wistar albino	both	24.5	20 mg, ip+22.7%lard	Placebo	[185]
Shea CM	Dahl/SS	rat		male	7	HS, 8% NaCl	LS, 0.3% NaCl	[186]
Singh K	STZ	rat	Wistar			50 mg/kg, ip	Placebo	[187]
Siri FM	STZ+RAL	rat	Wistar	female		60 mg/kg, iv	Placebo	[188]
Smart CD	uninephrectomized DOCA	mouse	C57BL/6J	male	10	100 mg, sc 3 wks	Sham operated+placebo	[189]
Soranno DE	bilateral R-IR	mouse	C57BL/6	female	8	25 min.	Sham operated	[190]
Soranno DE	bilateral R-IR	mouse	C57BL/6	male	8	25 min.	Sham operated	[191]
Soulié M	5/6 SNX	rat	SD	male	10		Sham operated	[192]
Suematsu Y	5/6 SNX	rat	SD	male			Sham operated	[193]
Sumida M	bilateral R-IR	mouse	C57BL/6	male	8	30 min.	Sham operated	[194]

Sun Z	uninephrectomized DOCA	rat	SD	male		35 mg/kg, sc once a week, 4 wks	Sham operated+placebo	[195]
Sung PH	double IR	rat	SD	male			Sham operated	[196]
Szymanski MK	LAD-MI	rat	Munich Wistar Fromter	male	12		Sham operated	[197]
Takahashi T	SHR+L-NAME	rat	SHR	male	17	50 mg/ml, 3 wks	Placebo	[198]
Tanaka S	5/6 SNX	mouse	129/Sv	male			Sham operated	[199]
Tate M	STZ+HFD	mouse	FVB/N	male	6	55 mg/kg, ip 3 days +42%fat	Placebo	[200]
Terker AS	LAD-MI	mouse	C57Bl/6	male	8		Sham operated	[201]
Tojo A	Dahl/SS	rat			6	HS, 8% NaCl	LS, 0.3% NaCl	[202]
Tracz MJ	bilateral R-IR	mouse			10	15 min.	Sham operated	[203]
Tran DT	STZ+HFD	mouse	C57BL/6J	male	7.5	90 mg/kg, ip +45%kcal fat	Placebo	[204]
Tykvartova T	ACF	rat	HanSD	male	8		Sham operated	[205]
Uchida L	5/6 SNX	rat	SD	male	8		Sham operated	[206]
Urbanek K	Dahl/SS	rat	HanSD	male	6	HS, 8% NaCl	LS, 0.3% NaCl	[207]
Vacková Š	ACF	rat		male	9		Sham operated	[208]
Vacková Š	ACF	rat	FHH	male			Sham operated FHL	[209]
van der Pol A	M-IR	mouse		male	17	60 min, 4wks	Sham operated	[210]
van Dokkum RP	LAD-MI	rat	Wistar	male			Sham operated	[211]
Verhulst A	ARF	rat	Wistar	male		0.2 mg/kg, per os 4 wks	Placebo	[212]
Verkaik M	5/6 SNX	mouse	C57BL/6	male	7		Sham operated	[213]
Vesely DL	GK	rat		both	10		Wistar	[214]
Wang BH	uninephrectomized DOCA	mouse	C57BL/6	male	6	6 wks	Sham operated, placebo	[215]
Wang D	uninephrectomized DOCA	rat	SD	male	8	50 mg, sc 8 wks	Sham operated, placebo	[216]
Wang F	uninephrectomized DOCA	rat	SD	male		50 mg, sc 3 wks	Sham operated, placebo	[217]
Wang J	bilateral R-IR	mouse	C57BL/6	male	8	35 min.	Sham operated	[218]
Wang J	bilateral R-IR	mouse	C57BL/6	male	8	30 min.	Sham operated	[219]
Wang J	LAD-MI	rat	SD	male			Sham operated	[220]
Wang N	ISO-HF	rat	SD	male		4 mg/kg, sc 10 days	Placebo	[221]
Wang YY	LAD-MI	rat	SPF	male			Sham operated	[222]
Watanabe H	Dahl/SS	rat			6	HS, 8% NaCl	LS, 0.3% NaCl	[223]

Watanabe K	TAC	mouse	C57BL/6	male	8		Sham operated	[224]
Watanabe R	LAD-MI	rat	SD	male	7		Sham operated	[225]
Wesseling M	TAC	mouse	C57BL/6J	both	9		Sham operated	[226]
Wiley DH	SHR	rat	SHR	both	8		WKY	[227]
Windt WA	5/6 SNX	rat	Wistar	male			Sham operated	[228]
Wu C	TAC	mouse	C57BL/6N	male	8		Sham operated	[229]
Wu CJ	5/6 SNX	rat	WKY	male			Sham operated	[230]
Wu J	UNX+ACF	rat	SD	male			Sham operated	[231]
Wu-Wong JR	5/6 SNX	rat	SD	male			Sham operated	[232]
Wu-Wong JR	5/6 SNX	rat	SD	male			Sham operated	[233]
Xiong Y	5/6 SNX	rat	Wistar	male	8		Sham operated	[234]
Xu X	3/4 SNX+ LAD-MI	rat	SD	male	8		Sham operated	[235]
Yamaguchi K	Dahl/SS	rat			5	HS, 2% NaCl	LS, 0.3% NaCl	[236]
Yamamoto E	SHR stroke-prone	rat	SD	male			WKY	[237]
Yang CC	5/6 SNX+Dox-HF	rat	SD	male			Sham operated+placebo	[238]
Yang CC	5/6 SNX+Dox-HF	rat	SD	male		7 mg/kg given at 4 separate time points within 20 days	Sham operated+placebo	[239]
Yang CC	5/6 SNX+LAD-MI	rat	SD	male			Sham operated	[240]
Yang CC	5/6 SNX	rat	WKY	male	9		Sham operated	[241]
Young SL	TAC	mouse	C57BL/6J	male	4		Sham operated	[242]
Yuen DA	5/6 SNX	rat	Fischer 344	male	8		Sham operated	[243]
Yurista SR	MI	rat	SD	male			Sham operated	[244]
Zeng S	5/6 SNX	rat	Wistar	male	8		Sham operated	[245]
Zhang MJ	UNX+ACF	rat	SD	male			Sham operated	[246]
Zhang Y	SHR	rat	SHR		8		WKY	[247]
Zhao Y	TAC	mouse	C57BL/6	male			Sham operated	[248]
Zheng G	ISO-HF	rat	SD	male	6	5 mg/kg, one week	Placebo	[249]
Zheng H	MI	rat	HanSD				Sham operated	[250]
Zheng H	MI	rat	SD	male			Sham operated	[251]
Zhou B	LAD-MI+SHR	rat	SHR	male	8		Sham operated	[252]
Zhou H	ISO-HF	rat	SD	male	6	5 mg/kg, sc one week	Placebo	[253]
Zhu X	LAD-MI	rat	Wistar	male	8		Sham operated	[254]

Zimmer DP	Dahl/SS	rat		male	HS, 8% NaCl	LS, 0.3% NaCl	[255]
Zou D	5/6 SNX+LAD-MI	rat	SD	male		Sham operated	[256]

2K1C – 2-kidney 1-clip; ACF – aorto-caval fistula; ARF – adenine-induced renal failure; $CoCl_2$-HF – cobalt dichloride-induced HF; Dahl/SR – Dahl Salt-Resistant; Dahl/SS – Dahl Salt-Sensitive; DOCA-salt – deoxycorticosterone acetate; double IR – acute heart and kidney ischemia-reperfusion; Dox-HF – doxorubicin-induced heart failure; FHH – Fawn-hooded hypertensive; FHL – Fawn-hooded low-pressure rats; GK – Goto-Kakizaki; HanSD – Hannover Sprague-Dawley; HFD – high fat diet; HS – high salt; ISO-HF – isoproterenol-induced heart failure; LAD-MI – left anterior descending coronary artery ligation; L-NAME – N(ω)-nitro-L-arginine methyl ester; LS – low salt; MI – coronary artery ligation; M-IR – myocardial ischemia-reperfusion; PAC – pulmonary artery constriction; RAL – renal artery ligation; Ren-2 Tg – (mRen2)27 transgenic; R-IR – renal ischemia-reperfusion injury; SD – Sprague-Dawley; SHR – spontaneous hypertensive rat; SNX – subtotal nephrectomy; SR – slow release; STZ – streptozotocin injected; TAC – transverse aortic constriction; UNX – unilateral nephrectomy; UUO – unilateral urinary obstruction; WD – western diet; WKY – Wistar Kyoto.

Supplementary. S4 Table. Results of meta-analysis.

**Parameter [unit] (effect size)**	**Animal model**	**Mean**	**Stand. error**	**p-value**	**No of trials /interventions***
**ACR (R, response ratio)**	5/6SNX	3.36	0.6	<0.0001	10
	5/6SNX+MI (LAD-MI)	2.10	0.42	=0.0002	4
	Dahl/SS	7.46	1.49	<0.0001	4
	MI (LAD-MI)	1.26	0.18	NS	10
	TAC	2.36	0.40	<0.0001	5
	uninephrectomized DOCA	3.47	0.51	<0.0001	5
**BUN [mg/dl] (D, difference in means)**	5/6SNX	41.92	2.67	<0.0001	36
	5/6SNX+Dox-HF	67.16	21.44	=0.0017	3
	5/6SNX+MI (LAD-MI)	49.71	14.48	=0.0006	3
	bilateral R-IR	111.93	11.73	<0.0001	10
	Dahl/SS	4.80	1.93	=0.012	6
	Dox-HF	58.29	12.81	<0.0001	3
	ISO-HF	12.47	6.19	=0.044	3
	MI (LAD-MI)	5.99	1.60	=0.0002	13
	STZ	23.63	5.51	<0.0001	5
**Cardiac fibrosis (R, response ratio)**	5/6SNX	2.43	0.34	<0.0001	19
	5/6SNX+MI (LAD-MI)	2.28	0.96	NS	3
	MI (LAD-MI)	2.41	0.46	<0.0001	7
	SHR	3.00	0.24	<0.0001	3
	SHR-stroke prone	2.62	0.81	=0.002	3
	STZ	3.60	0.65	<0.0001	6
	uninephrectomized DOCA	4.07	1.01	<0.0001	8
**dP/dt_{max} [mmHg/s] (D, difference in means)**	5/6SNX	-1190.75	778.35	NS	7
	ISO-HF	-4840.85	1061.94	<0.0001	4
	MI (LAD-MI)	-2218.43	292.04	<0.0001	18
	STZ	-1735.47	426.47	<0.0001	4
**dP/dt_{min} [mmHg/s] (D, difference in means)**	5/6SNX	-2310.32	669.56	=0.0006	5
	5/6SNX+MI (LAD-MI)	-2950.62	983.89	=0.0027	3
	ISO-HF	-3967.19	1247.40	<0.0001	3
	MI (LAD-MI)	-1896.00	172.56	<0.0001	14
	STZ	-1192.17	227.10	<0.0001	4
**EF [%] (D, difference in means)**	5/6SNX	-5.56	2.03	=0.006	28
	5/6SNX+Dox-HF	-15.66	4.43	=0.0004	6
	5/6SNX+MI (LAD-MI)	-40.65	3.47	<0.0001	9
	bilateral R-IR	-5.88	2.44	=0.016	3
	Dahl/SS	-2.66	2.27	NS	7
	ISO-HF	-23.74	5.15	<0.0001	6
	MI (LAD-MI)	-28.44	4.28	<0.0001	27
	M-IR	-21.79	4.50	<0.0001	4
	TAC	-19.67	5.59	=0.0004	12
	uninephrectomized DOCA	0.66	3.74	NS	6
	5/6SNX	-1.85	1.90	NS	21
	5/6SNX+MI (LAD-MI)	-25.17	2.49	<0.0001	8

**FS [%] (D, difference in means)**	ACF	-18.72	1.18	<0.0001	4
	bilateral R-IR	-0.61	2.20	NS	10
	Dahl/SS	-13.51	4.01	=0.0007	10
	ISO-HF	-19.23	6.97	=0.006	6
	MI (LAD-MI)	-25.10	0.45	<0.0001	27
	Ren-2Tg+ACF	-15.24	3.59	<0.0001	3
	STZ	-16.06	3.71	<0.0001	4
	TAC	-18.35	3.16	<0.0001	6
	uninephrectomized DOCA	-0.23	1.53	NS	3
**GFR (R, response ratio)**	5/6SNX	0.41	0.12	=0.003	9
	5/6SNX+MI (LAD-MI)	0.28	0.14	=0.012	4
	ACF	0.74	0.09	=0.018	7
	bilateral R-IR	0.08	0.11	NS	4
	Dahl/SS	0.64	0.11	=0.01	4
	MI (LAD-MI)	0.77	0.05	<0.0001	14
	Ren-2Tg	1.16	0.03	<0.0001	3
	Ren-2Tg+ACF	0.96	0.08	NS	3
**Glomerulosclerosis index score (R, response ratio)**	5/6SNX	2.49	0.38	<0.0001	12
	Dahl/SS	8.30	1.52	<0.0001	5
	MI (LAD-MI)	2.19	0.83	=0.037	3
	uninephrectomized DOCA	5.48	2.10	<0.0001	5
**Kidney hypertrophy (R, response ratio)**	5/6SNX	1.34	0.12	=0.0009	8
	5/6SNX+MI (LAD-MI)	1.46	0.07	<0.0001	3
	ACF	1.01	0.03	NS	7
	Dahl/SS	1.63	0.26	=0.002	9
	Goto-Kakizaki	1.23	0.02	<0.0001	3
	ISO-HF	1.12	0.04	=0.002	4
	MI (LAD-MI)	1.03	0.02	NS	16
	Ren-2Tg	1.06	0.05	NS	6
	Ren-2Tg+ACF	1.01	0.01	NS	6
	SHR	1.05	0.01	<0.0001	3
	SHR-stroke prone	1.57	0.37	NS	3
	STZ	1.70	0.16	<0.0001	7
	TAC	0.98	0.08	NS	3
	uninephrectomized DOCA	2.15	0.18	<0.0001	9
**LVEDd [mm] (D, difference in means)**	5/6SNX	0.36	0.19	NS	18
	5/6SNX+Dox-HF	0.19	0.19	NS	3
	5/6SNX+MI (LAD-MI)	1.71	1.05	NS	3
	ACF	4.23	0.85	<0.0001	6
	Dahl/SS	0.64	0.32	=0.047	9
	MI (LAD-MI)	1.73	0.20	<0.0001	18
	Ren-2Tg	-0.93	0.48	=0.05	3
	Ren-2Tg+ACF	4.03	0.41	<0.0001	3
	TAC	0.41	0.20	=0.04	7
**LVESd [mm] (D, difference in means)**	5/6SNX+MI (LAD-MI)	2.76	1.27	=0.029	3
	5/6SNX+Dox-HF	1.04	0.52	=0.045	3
	ACF	3.26	0.76	<0.0001	3
	Dahl/SS	0.83	0.60	NS	5
	MI (LAD-MI)	2.75	0.24	<0.0001	17
	Ren-2Tg+ACF	3.54	0.51	<0.0001	3
	TAC	0.59	0.13	<0.0001	8
**LVEDP [mmHg] (D, difference in means)**	5/6SNX	3.67	0.57	<0.0001	10
	5/6SNX+MI (LAD-MI)	5.70	0.84	<0.0001	3
	ACF	5.63	1.05	<0.0001	3
	bilateral R-IR	0.38	0.31	NS	4
	Dahl/SS	8.21	3.30	=0.01	3
	ISO-HF	12.75	0.92	<0.0001	4
	MI (LAD-MI)	11.51	1.60	<0.0001	20
	STZ	4.83	1.92	=0.01	3
	uninephrectomized DOCA	5.59	3.00	=0.06	3
	5/6SNX	17.93	6.35	=0.005	6

**LVESP [mmHg] (D, difference in means)**	ISO-HF (rat)	-31.09	1.50	<0.0001	4
	MI (LAD-MI)	-13.90	2.22	<0.0001	9
**Myocardial hypertrophy (R, response ratio)**	2K1C	1.33	0.09	=0.0001	6
	5/6SNX	1.23	0.03	<0.0001	41
	5/6SNX+MI (LAD-MI)	1.16	0.04	<0.0001	9
	ACF	1.62	0.15	<0.0001	14
	ARF	1.35	0.05	<0.0001	5
	bilateral R-IR	1.16	0.05	=0.0006	8
	Dahl/SS	1.49	0.11	<0.0001	17
	ISO-HF	1.36	0.04	<0.0001	8
	MI (LAD-MI)	1.25	0.03	<0.0001	37
	Ren-2Tg	1.21	0.05	<0.0001	10
	Ren-2Tg+ACF	1.65	0.05	<0.0001	8
	SHR	1.20	0.08	=0.009	4
	SHR-stroke prone	1.56	0.12	<0.0001	4
	STZ	1.56	0.19	=0.0003	8
	TAC	1.52	0.07	<0.0001	11
	unilateral R-IR	1.18	0.03	<0.0001	4
	uninephrectomized DOCA	1.33	0.02	<0.0001	16
	UNX+ACF	1.53	0.09	<0.0001	4
**SBP [mmHg] (D, difference in means)**	2K1C	77.47	8.55	<0.0001	6
	5/6SNX	39.93	5.20	<0.0001	37
	5/6SNX+MI (LAD-MI)	10.04	8.14	NS	6
	ARF	14.58	14.37	NS	3
	Dahl/SS	62.76	9.25	<0.0001	17
	MI (LAD-MI)	-6.22	1.70	=0.0003	18
	SHR	68.66	18.13	=0.0002	4
	SHR-stroke prone	110.84	11.68	<0.0001	3
	STZ	17.14	11.46	NS	6
	TAC	-0.66	1.63	NS	5
	uninephrectomized DOCA	45.36	4.15	<0.0001	13
**S-Cre [mg/dl] (D, difference in means)**	2K1C	0.26	0.07	=0.0001	7
	5/6SNX	0.56	0.04	<0.0001	55
	5/6SNX+MI (LAD-MI)	0.78	0.13	<0.0001	9
	5/6SNX+Dox-HF	1.03	0.17	<0.0001	6
	ACF	0.01	0.01	NS	3
	ARF	2.42	0.96	=0.012	5
	bilateral R-IR	1.33	0.18	<0.0001	11
	Dahl/SS	0.09	0.02	<0.0001	11
	Dox-HF	0.57	0.18	=0.0013	4
	ISO-HF	0.18	0.18	NS	5
	MI (LAD-MI)	0.13	0.01	<0.0001	28
	M-IR	0.58	0.27	=0.03	4
	STZ	0.43	0.12	=0.0005	6
	unilateral R-IR	0.70	0.47	NS	4
	uninephrectomized DOCA	0.23	0.07	=0.0008	3
**Renal fibrosis (R, response ratio)**	5/6SNX	4.26	0.79	<0.0001	11
	5/6SNX+(LAD)MI	2.98	1.09	=0.003	3
	Dahl/SS	2.28	0.38	<0.0001	5
	ISO-HF	6.17	1.91	<0.0001	4
	MI (LAD-MI)	2.19	0.45	=0.0001	8
	STZ	2.42	0.43	=0.0001	3
	uninephrectomized DOCA	13.88	11.90	=0.002	3
**UAlbEx [mg/24h] (D, difference in means)**	5/6SNX	65.71	1.78	<0.0001	11
	ACF	5.14	2.50	=0.04	3
	Dahl/SS	88.45	42.64	=0.04	3
	MI (LAD-MI)	0.35	0.12	=0.004	5
**UProtEx [mg/24h] (D, difference in means)**	5/6SNX	136.09	11.26	<0.0001	18
	5/6SNX+MI (LAD-MI)	46.05	3.76	<0.0001	7
	Dahl/SS	512.59	145.12	<0.0001	3
	MI (LAD-MI)	12.46	4.42	=0.005	13
	SHR-stroke prone	61.40	26.04	=0.018	5
	UNX+MI	83.13	58.38	NS	3
	5/6SNX (rat)	-35.11	6.33	<0.0001	26

**BW [g] (D, difference in means)**	5/6SNX (mouse)	-4.48	1.99	=0.024	4
	ACF (rat)	6.87	10.53	NS	11
	ARF (rat)	-100.03	44.05	=0.02	5
	Dahl/SS (rat)	-55.56	9.59	<0.0001	19
	ISO-HF (rat)	-37.75	6.57	<0.0001	4
	MI (LAD-MI) (mouse)	1.44	0.85	NS	15
	MI (LAD-MI) (rat)	-9.97	9.03	NS	18
	Ren-2Tg (rat)	46.33	27.58	NS	7
	Ren-2Tg+ACF (rat)	-29.18	11.22	=0.009	4
	SHR (rat)	-10.86	16.83	NS	3
	STZ (rat)	-99.37	15.91	<0.0001	9
	TAC (mouse)	0.05	0.37	NS	8
	unilateral R-IR (mouse)	-1.21	0.44	=0.006	3
	uninephrectomized DOCA (rat)	-59.13	6.5	<0.0001	5
	uninephrectomized DOCA (mouse)	-2.76	0.43	<0.0001	5

* - at least three interventions must have been included into analysis, in relation to individual parameter and animal model. Mostly, the significance of the overall result, with regard to individual parameter, was not influenced by any of the selected studies included into analysis.The exception concerned the alternations in EF parameter that were observed in ARF model, LVESd – in 5/6SNX or Ren-2Tg animals, S-Cre – in TAC subjects, and LVEDP – in uninephrectomized animals exposed to coronary artery ligation. 2K1C – 2-kidney 1-clip; ACF – aorto-caval fistula; ACR – albumin to creatinine urinary excretion ratio; ARF – adenine-induced renal failure; BUN – blood urea nitrogen; BW – body weight; DOCA-salt – deoxycorticosterone acetate; Dox-HF – doxorubicin-induced heart failure; dP/dt_{max} – maximal rate of pressure increase; dP/dt_{min} – maximal rate of pressure decrease; EF – left ventricle ejection fraction; FS – fractional shortening; GFR – glomerular filtration rate; GK – Goto-Kakizaki; ISO-HF – isoproterenol-induced HF; LAD-MI – left anterior descending coronary artery ligation; LVEDd – left ventricular end-diastolic diameter; LVEDP – left ventricular end-diastolic pressure; LVESd – left ventricular entricle end-systolic diameter; LVESP – left ventricular end-systolic pressure; Ren-2 Tg – (mRen2)27 transgenic; R-IR – renal ischemia-reperfusion; SBP – systolic blood pressure; S-Cre – serum creatinine; SHR – spontaneous hypertensive rat; SNX – subtotal nephrectomy; STZ – streptozotocin injected; TAC – transverse aortic constriction; UAlbEx – urinary albumin excretion; UNX – unilateral nephrectomy; UProtEx – urinary protein excretion; UUO – unilateral urinary obstruction.

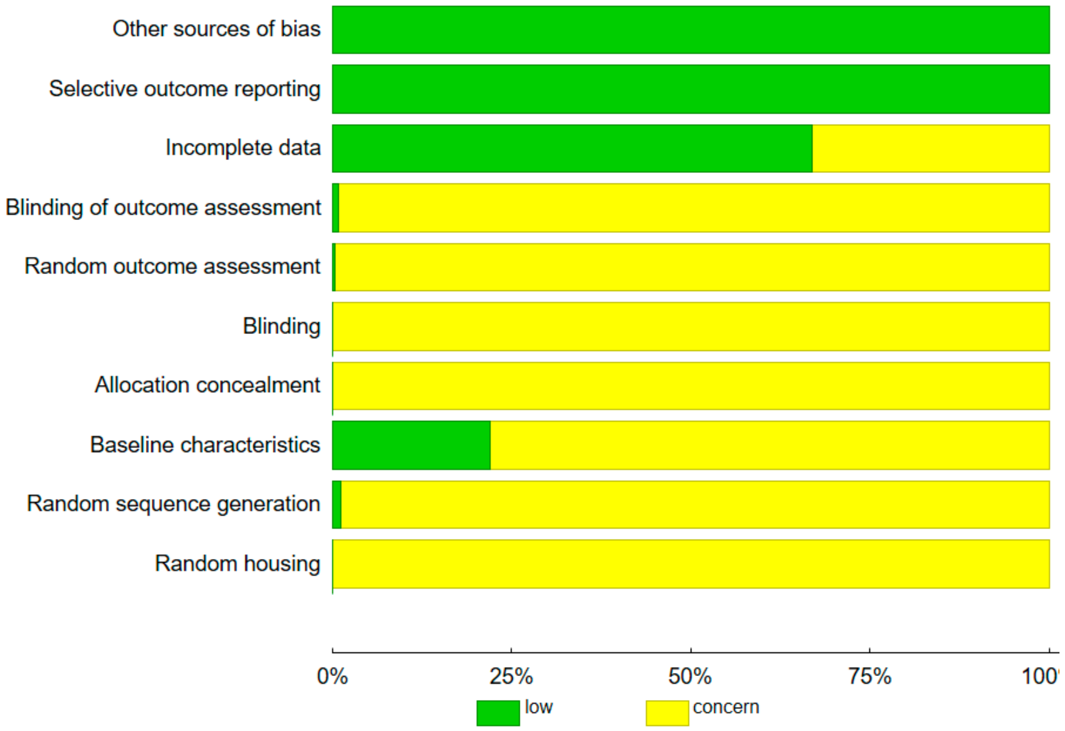

Supplementary. Figure S1. Summary of risk of bias according to the SYRCLE RoB strategy (Hooijmans et al., 2014). Over half of protocols (146/251; 58.2 %) performed random assignment of animals to study groups; however, no detailed description of the method used to generate the allocation sequence was provided (unclear risk of bias – concern). Only three described the randomization process (low risk of bias). The remaining protocols did not discuss the randomization process (unclear risk of bias). The blinding process was described in 94 of the 251 studies (37.4%). The authors mainly mentioned that echocardiographic, histomorphometric data were collected under blinded conditions during the whole experiment; this blinding also concerned the statistical analyses. No detailed information was given regarding the procedure of blinding the researchers to the animal groups (unclear risk of bias – concern). Two papers gave a more detailed description of blinding the outcome assessment. None of the remaining protocols mentioned blinding (unclear risk

of bias – concern). In addition, 21.9% of papers described assessments of baseline characteristics in experimental and control groups before starting an experiment (low risk of bias). In 33.1% of papers, the number of subjects in each group that finished the experiment was not reported (unclear risk of bias).

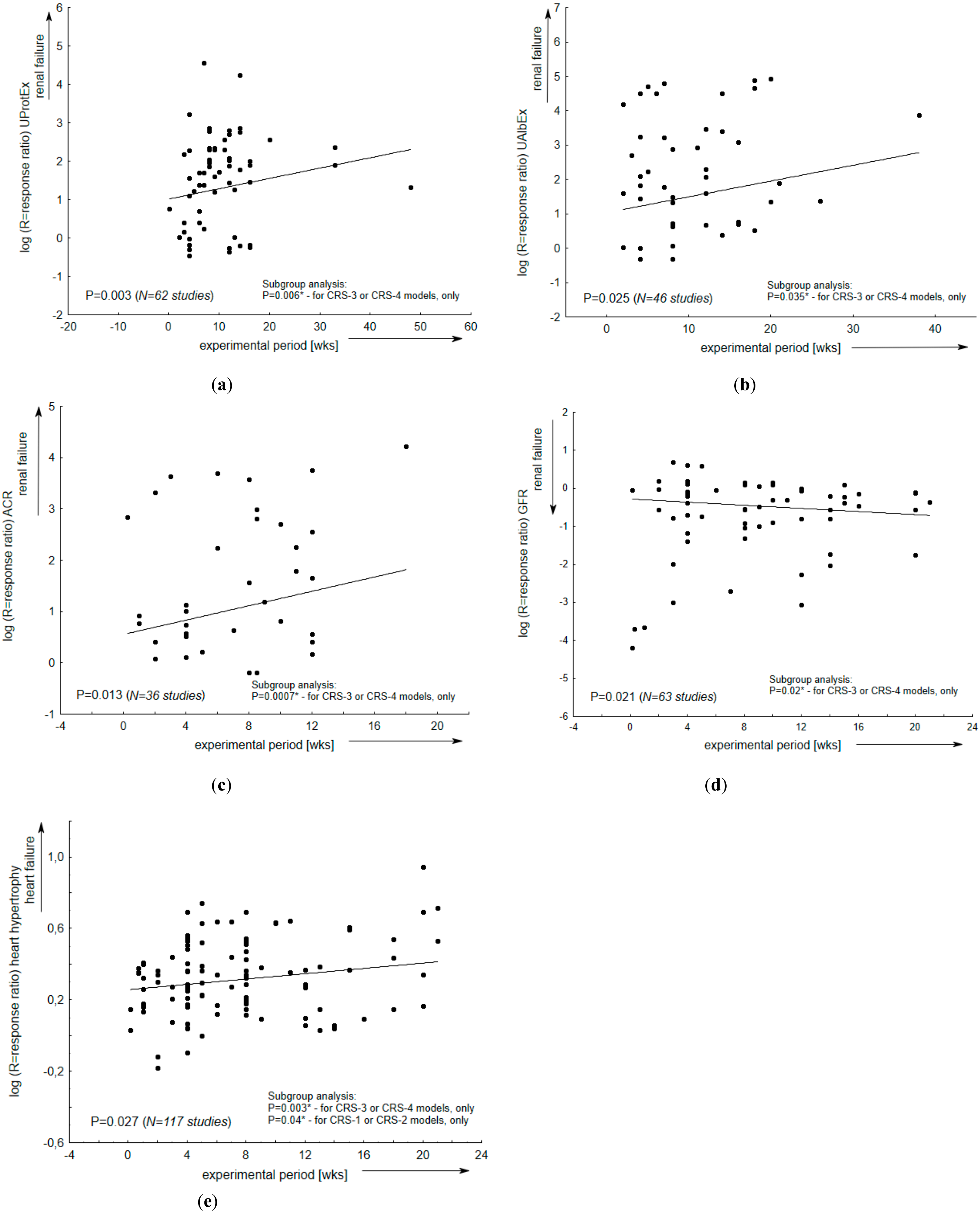

Supplementary. Figure S2. The impact of experimental duration (weeks) on the worsening of selected renal and cardiac parameters in selected CRS animal models. Meta-regression lines have been fitted to indicate effect size expressed as response ratio (R). (**a**) urinary protein excretion ratio (UProtEx, mg/24h); (**b**) urinary albumin excretion ratio (UAlbEx, mg/24h); (**c**) urinary albumin to creatinine excretion ratio (ACR); (**d**) – glomerular filtration rate (GFR); (**e**) cardiac hypertrophy. N – total number of comparisons.

Section and Topic	Item #	Checklist item	Location where item is reported
**TITLE**			
Title	1	Identify the report as a systematic review.	p.1
**ABSTRACT**			
Abstract	2	See the PRISMA 2020 for Abstracts checklist.	p.1
**INTRODUCTION**			
Rationale	3	Describe the rationale for the review in the context of existing knowledge.	p.1-2
Objectives	4	Provide an explicit statement of the objective(s) or question(s) the review addresses.	p.2
**METHODS**			
Eligibility criteria	5	Specify the inclusion and exclusion criteria for the review and how studies were grouped for the syntheses.	p.2-4, Suppl. Material
Information sources	6	Specify all databases, registers, websites, organisations, reference lists and other sources searched or consulted to identify studies. Specify the date when each source was last searched or consulted.	p.2-4, Fig.1
Search strategy	7	Present the full search strategies for all databases, registers and websites, including any filters and limits used.	p.2-4, Fig.1
Selection process	8	Specify the methods used to decide whether a study met the inclusion criteria of the review, including how many reviewers screened each record and each report retrieved, whether they worked independently, and if applicable, details of automation tools used in the process.	p.2-4, Suppl. Material
Data collection process	9	Specify the methods used to collect data from reports, including how many reviewers collected data from each report, whether they worked independently, any processes for obtaining or confirming data from study investigators, and if applicable, details of automation tools used in the process.	p.3-4
Data items	10a	List and define all outcomes for which data were sought. Specify whether all results that were compatible with each outcome domain in each study were sought (e.g. for all measures, time points, analyses), and if not, the methods used to decide which results to collect.	p.2, Suppl. Material
	10b	List and define all other variables for which data were sought (e.g. participant and intervention characteristics, funding sources). Describe any assumptions made about any missing or unclear information.	p.2, Suppl. Material
Study risk of bias assessment	11	Specify the methods used to assess risk of bias in the included studies, including details of the tool(s) used, how many reviewers assessed each study and whether they worked independently, and if applicable, details of automation tools used in the process.	p.3-4
Effect measures	12	Specify for each outcome the effect measure(s) (e.g. risk ratio, mean difference) used in the synthesis or presentation of results.	p.3-4, Suppl. Material
Synthesis methods	13a	Describe the processes used to decide which studies were eligible for each synthesis (e.g. tabulating the study intervention characteristics and comparing against the planned groups for each synthesis (item #5)).	p.3-4, S3 Table
	13b	Describe any methods required to prepare the data for presentation or synthesis, such as handling of missing summary statistics, or data conversions.	p.3-4, Suppl. Material
	13c	Describe any methods used to tabulate or visually display results of individual studies and syntheses.	p.3-4, Suppl. Material
	13d	Describe any methods used to synthesize results and provide a rationale for the choice(s). If meta-analysis was performed, describe the	p.3-4, Suppl.

Section and Topic	Item #	Checklist item	Location where item is reported
		model(s), method(s) to identify the presence and extent of statistical heterogeneity, and software package(s) used.	Material
	13e	Describe any methods used to explore possible causes of heterogeneity among study results (e.g. subgroup analysis, meta-regression).	p.3-4, Suppl. Material
	13f	Describe any sensitivity analyses conducted to assess robustness of the synthesized results.	p.3-4, Suppl. Material
Reporting bias assessment	14	Describe any methods used to assess risk of bias due to missing results in a synthesis (arising from reporting biases).	p.3-4, Suppl. Material
Certainty assessment	15	Describe any methods used to assess certainty (or confidence) in the body of evidence for an outcome.	p. 3-4
**RESULTS**			
Study selection	16a	Describe the results of the search and selection process, from the number of records identified in the search to the number of studies included in the review, ideally using a flow diagram.	Fig.1
	16b	Cite studies that might appear to meet the inclusion criteria, but which were excluded, and explain why they were excluded.	p.4
Study characteristics	17	Cite each included study and present its characteristics.	S3 Table
Risk of bias in studies	18	Present assessments of risk of bias for each included study.	p.4, Fig.S1
Results of individual studies	19	For all outcomes, present, for each study: (a) summary statistics for each group (where appropriate) and (b) an effect estimate and its precision (e.g. confidence/credible interval), ideally using structured tables or plots.	Table 1, Fig.2, Fig.3. S4 Table
Results of syntheses	20a	For each synthesis, briefly summarise the characteristics and risk of bias among contributing studies.	p.4, Fig S1
	20b	Present results of all statistical syntheses conducted. If meta-analysis was done, present for each the summary estimate and its precision (e.g. confidence/credible interval) and measures of statistical heterogeneity. If comparing groups, describe the direction of the effect.	Table 1, Fig.2, Fig.3. S4 Table
	20c	Present results of all investigations of possible causes of heterogeneity among study results.	p.4, p.9
	20d	Present results of all sensitivity analyses conducted to assess the robustness of the synthesized results.	S2 Table
Reporting biases	21	Present assessments of risk of bias due to missing results (arising from reporting biases) for each synthesis assessed.	S1 Table
Certainty of evidence	22	Present assessments of certainty (or confidence) in the body of evidence for each outcome assessed.	p. 4, 9
**DISCUSSION**			
Discussion	23a	Provide a general interpretation of the results in the context of other evidence.	p.13-20
	23b	Discuss any limitations of the evidence included in the review.	p.22-23
	23c	Discuss any limitations of the review processes used.	p.22-23
	23d	Discuss implications of the results for practice, policy, and future research.	p.20-21

Section and Topic	Item #	Checklist item	Location where item is reported
**OTHER INFORMATION**			
Registration and protocol	24a	Provide registration information for the review, including register name and registration number, or state that the review was not registered.	p.23-24
	24b	Indicate where the review protocol can be accessed, or state that a protocol was not prepared.	p.23-24
	24c	Describe and explain any amendments to information provided at registration or in the protocol.	/
Support	25	Describe sources of financial or non-financial support for the review, and the role of the funders or sponsors in the review.	p.23-24
Competing interests	26	Declare any competing interests of review authors.	p.23-24
Availability of data, code and other materials	27	Report which of the following are publicly available and where they can be found: template data collection forms; data extracted from included studies; data used for all analyses; analytic code; any other materials used in the review.	p.23-24

From: Page MJ, McKenzie JE, Bossuyt PM, Boutron I, Hoffmann TC, Mulrow CD, et al. The PRISMA 2020 statement: an updated guideline for reporting systematic reviews. BMJ 2021;372:n71. doi: 10.1136/bmj.n71. This work is licensed under CC BY 4.0. To view a copy of this license, visit https://creativecommons.org/licenses/by/4.0/
